# Quantifying surface tension and viscosity in biomolecular condensates by FRAP-ID

**DOI:** 10.1016/j.bpj.2024.07.043

**Published:** 2024-08-08

**Authors:** Andreas Santamaria, Stephanie Hutin, Christine M. Doucet, Chloe Zubieta, Pierre-Emmanuel Milhiet, Luca Costa

**Affiliations:** 1 Center for Structural Biology (CBS), CNRS, INSERM, Montpellier University, Montpellier, France; 2 Laboratoire de Physiologie Cellulaire et Végétale, Université Grenoble-Alpes, CNRS, CEA, INRAE, IRIG-DBSCI, Grenoble, France

## Abstract

Many proteins with intrinsically disordered regions undergo liquid-liquid phase separation under specific conditions in vitro and in vivo. These complex biopolymers form a metastable phase with distinct mechanical properties defining the timescale of their biological functions. However, determining these properties is nontrivial, even in vitro, and often requires multiple techniques. Here we report the measurement of both viscosity and surface tension of biomolecular condensates via correlative fluorescence microscopy and atomic force microscopy (AFM) in a single experiment (fluorescence recovery after probe-induced dewetting, FRAP-ID). Upon surface tension evaluation via regular AFM-force spectroscopy, controlled AFM indentations induce dry spots in fluorescent condensates on a glass coverslip. The subsequent rewetting exhibits a contact line velocity that is used to quantify the condensed-phase viscosity. Therefore, in contrast with fluorescence recovery after photobleaching (FRAP), where molecular diffusion is observed, in FRAP-ID fluorescence recovery is obtained through fluid rewetting and the subsequent morphological relaxation. We show that the latter can be used to cross-validate viscosity values determined during the rewetting regime. Making use of fluid mechanics, FRAP-ID is a valuable tool to evaluate the mechanical properties that govern the dynamics of biomolecular condensates and determine how these properties impact the temporal aspects of condensate functionality.

## Graphical abstract

**Figure:**
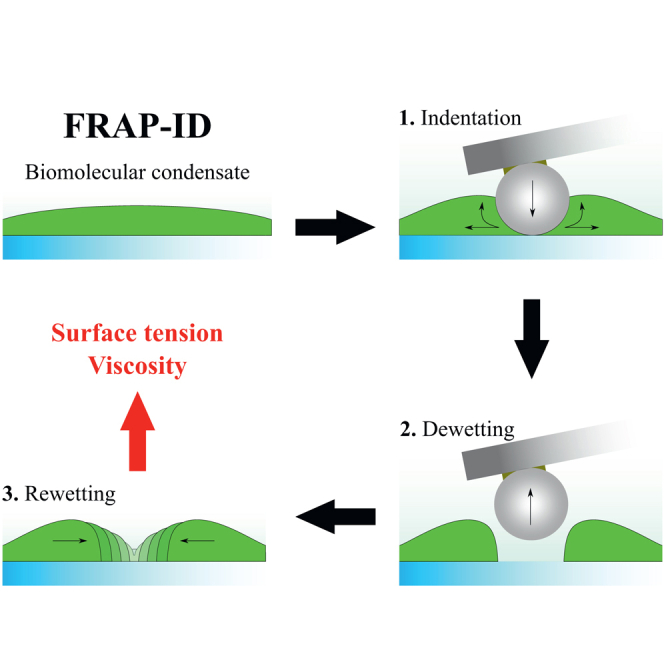

## Significance

Biomolecular condensates, resulting from the liquid-liquid phase separation of proteins, exhibit distinct surface tension and viscosity defining the timescale of their biological functions. Making use of fluid mechanics, these parameters are determined using a method that we named fluorescence recovery after probe-induced dewetting, FRAP-ID. Upon deposition onto a glass coverslip, fluorescent condensates are indented with a micrometric probe, leading to the formation of dry spots. The subsequent fluorescence recovery, characterized by fluid rewetting and morphological relaxation, is used to evaluate viscosity and surface tension.

## Introduction

Biomolecular condensates, formed by liquid-liquid phase separation (LLPS) of macromolecules (1,2,3) such as proteins, DNA, lipids, and glycogen, are essential for subcellular compartmentalization via the formation of membrane-less organelles (4). Examples of biomolecular condensates include the nucleolus and actin-mediated structures (5,6,7). These structures are believed to play an essential role by increasing local concentration of the partitioning molecules (4). Additionally, dysfunctions related to LLPS formation, dynamics, and associated mechanical properties, due to transitions to a solid/gel state for instance, are often related to the onset of pathologies (4,8,9,10,11,12,13,14). In this framework, properties such as viscosity (*η*) and surface tension (*γ*) between dilute and condensed phases, which govern LLPS structure and mechanics (15,16), are key parameters that need to be quantified in order to investigate condensate dynamics (e.g., droplet fusion), function, aging, molecular recruitment, diffusive processes, and condensate internal organization (17,18,19,20,21,22). Fluorescence recovery after photobleaching (FRAP) is the technique widely used to gain insights into biomolecular condensate fluidity, qualitatively estimating the diffusion coefficient of the condensed-phase component in vitro and in vivo (6,9,18,23,24,25,26,27,28,29,30,31). However, interpreting FRAP results (25,30) can be complex, especially for multicomponent condensates whose different size or nature results in multiple recovery velocities or static behavior. Passive microrheology is another technique used to determine the viscosity of the condensates and employs fluorescence microscopy to track the movement of fluorescent beads (usually ranging from 20 to 500 nm in diameter and functionalized with polyethylene glycols (30)), embedded within condensates (17,30,31,32,33,34,35). Applying the Stokes-Einstein equation allows for the evaluation of reliable viscosity values, provided that beads, located at a considerable distance from the edges of the droplets, undergo Brownian motion, therefore requiring the preparation of large droplets, which may be challenging even in vitro (17). Factors such as functionalization of the beads, their size, and the presence of heterogeneous micro- and nanodomains introduce additional parameters that contribute to the complexity of data interpretation (30,36). Active microrheology provides an attractive and more versatile alternative to these techniques via the use of optically trapped beads to measure condensate viscosity (37). Previous studies have demonstrated that atomic force microscopy (AFM) may be employed to investigate polymeric and biomolecular condensates in the liquid phase (15,38,39) or, more generally, liquid droplets and particles (40). Rheological properties can be obtained upon droplet confinement between the substrate and a micrometric spherical (colloidal) AFM probe, assuming that the system is at equilibrium at small contact angles. This approach reveals, unsurprisingly, consistently different viscoelastic values across the frequency spectrum (15). In contrast, conventional AFM quasi-static indentation cycles provide values under stationary conditions, as demonstrated in (39). The second important parameter to be determined is the surface tension of the sample. This can be assessed with optical tweezers (OT) (41), which trap fluorescent particles within droplets and subject them to an oscillatory trajectory. Monitoring the condensate response enables the evaluation of both surface tension and rheological behavior. However, optical traps are not suitable for studying droplets formed by thermoresponsive biomolecules, due to the high laser power employed, nor for condensates with high surface tension, as OT measurements are restricted to piconewton-range forces (15).

**Figure 1:**
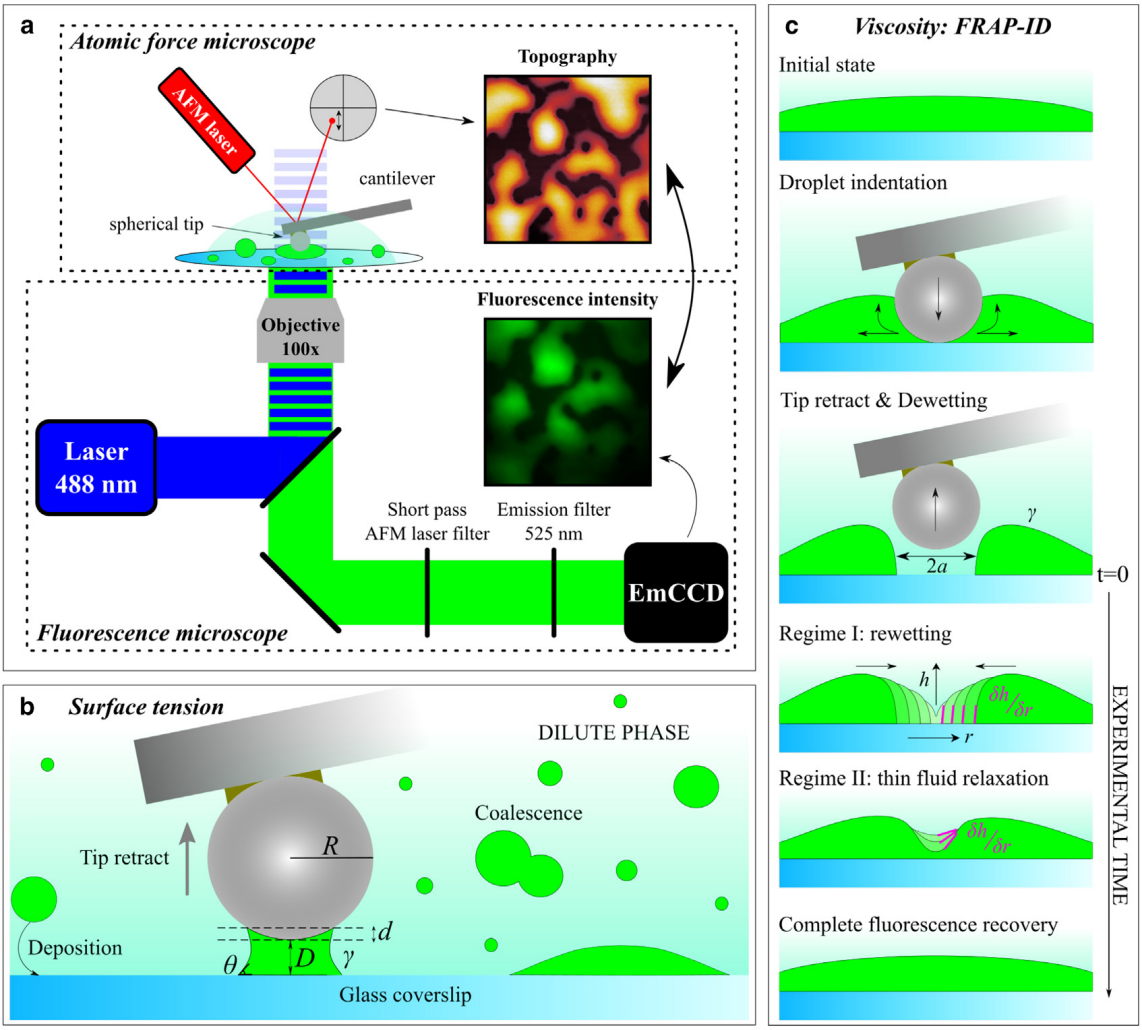
(*a*) Correlative AFM-epifluorescence microscopy setup. (*b*) Pictorial representation of a liquid droplet confined between the AFM colloidal probe with radius *R* and the glass substrate, and exerting an attractive force on the retracting probe. The force is used to assess the condensate surface tension (*γ*). (*c*) Sketch reporting the different phases of the FRAP-ID method. The formation of a dry spot (*top*) within the condensed phase (*green*) is tracked until complete rewetting (regime I). The contact line speed during this process is monitored through epifluorescence to determine the viscosity (*η*). Subsequently, complete fluorescence recovery is obtained through a fluid relaxation, whose lubrication analysis leads once again to η/γ, cross-validating values obtained from regime I.

The inverse capillary velocity, defined by the ratio η/γ, can be determined by monitoring the coalescence between two droplets (2,5,12,17,18,30,32,42). The characteristic fusion time (*τ*) is determined by monitoring the evolution of an elliptic area encompassing two droplets into a circular area with a single droplet, the final stage of the fusion event. The inverse capillary velocity acts as proportionality constant between *τ* and the droplet length scale, enabling the evaluation of *γ*, after previous determination of *η* by microrheology. Mainly used for micrometric and optically visible droplets, these experiments may require surface functionalization or the use of OT to avoid wetting or induce droplet fusion (24,28,29,31). Additionally, the determination of the elliptic area can be technically challenging (30), particularly in scenarios involving high surface tension in the range of millinewtons per meter or low viscosity, where accurately characterizing the rapid fusion process becomes difficult. In this manuscript, we propose a straightforward method that employs correlative and simultaneous atomic force and fluorescence microscopies (43,44,45,46,47,48,49) to determine *γ* and *η* of fluorescently labeled biomolecular condensates in a single experiment in vitro. Initially, the condensate is confined between a colloidal AFM probe and the substrate (see experimental setup shown in Fig. 1 *a*), whose mutual adhesive force allows us to determine the surface tension of the condensate (50) (Fig. 1 *b*). Small droplets, with size inferior to the diffraction limit, can be characterized in this way, in contrast to the techniques previously described that require large droplets. Subsequently, in a scenario where multiple droplets have wetted the substrate, covering large micrometric regions, dry spots are formed within these areas by applying an AFM force higher than 10–20 nN. Recording the fluorescent mass flow during rewetting until hole collapse (Fig. 1 *c*) enables the evaluation of the condensate viscosity (51,52). We name this technique FRAP-ID (fluorescence recovery after probe-induced dewetting). As a case of study, we use the prion-like domain of the EARLY FLOWERING 3 (ELF3) protein, tagged with Green Fluorescent Protein (GFP), undergoing LLPS. The unstructured prion-like domain present in ELF3 is responsible for driving condensate formation, in vitro and in vivo, in a temperature- and pH-dependent manner (44,53), and provides a robust and easily manipulated model system for these studies. Our results are then compared with data generated using passive microrheology, coalescence monitoring, and FRAP. The comparison reveals strong agreement with the obtained results. The detailed analysis of our methodology reveals the capability to evaluate surface tension as low as a few micronewtons per meter and viscosity down to 1 Pa · s. Further improvements are achievable by tuning the cantilever spring constant and adjusting the AFM probe size.

## Materials and methods

### Surface tension (*γ*)

**Figure 2:**
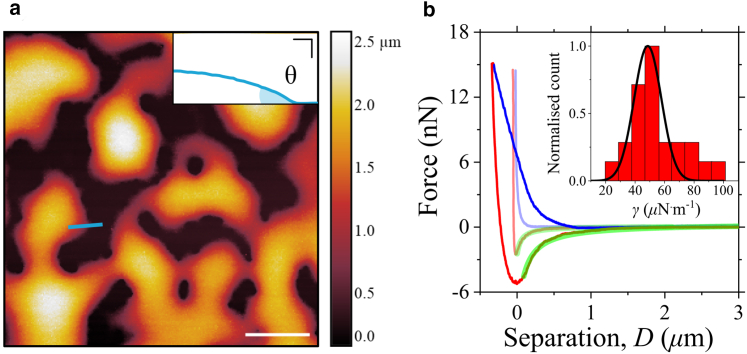
(*a*) AFM topography image of GFP-tagged ELF3 biomolecular condensates wetting a glass coverslip. Scale bar, 10 *μ*m. The static contact angle (*θ*), shown in the inset (scale bars on both *x* and *y* axis = 1 *μ*m), is determined along the profile (*light blue*) in the AFM image. (*b*) Two indentation cycles, performed with a colloidal probe (*R* = 2.5 *μ*m) onto different droplets, are shown with both approach (*blue*) and retract (*red*). Indentations exhibit different hysteresis, reflecting the different size of the droplets. Retract curves are fitted (*green*) using Eq. 1, providing the droplet surface tension, whose distribution and the associated Gaussian fit (*black*) are shown in the inset.

By means of epifluorescence, single droplets can be aligned below the AFM probe. Using AFM-force spectroscopy (AFM-FS) with moderate force (<10–20 nN), droplets can be sequentially confined between the AFM probe and the glass coverslip, acting as a concave meniscus that leads to the adhesive attraction of the solid surfaces (Fig. 1 *b*). Such attractive force is directly proportional to *γ*, and force-versus-distance indentation cycles exhibit a hysteretic behavior (38). In the case of a colloidal probe of radius *R*, the attractive force measured while retracting the probe can be expressed as follows (50):

(1) $$F\left(D\right)=-\frac{4\pi R\gamma \;\cos\;\theta }{1+\frac{D}{d}}\text{,}$$

where *θ* is the static contact angle at the three-phase boundary (substrate-condensed phase-dilute phase), *D* is the probe-substrate distance, and *d* is the height of the spherical probe cap that is wetted by the condensed phase. Eq. 1 holds for *R* ≫ *d* and therefore is applicable for cases in which droplets are much smaller than the colloidal probe. Fig. 1 *b* shows these parameters, alongside a schematic representing a droplet of the condensed phase (depicted in *bright green*) confined between AFM probe and substrate, immersed in the dilute phase (*light green*). *θ* is evaluated from AFM morphological images acquired using sharp pyramidal AFM probes with R≈ 8 nm (Figs. 2 *a* and S1 *a*). The estimation of the contact angle and condensate height derived from epifluorescence maps, expressed in nm/counts (Fig. 1 *a*), are both calibration routines performed with such a sharp pyramidal probe before assessing surface tension and viscosity using colloidal probes in an FRAP-ID experiment. With accurate calibrations, multiple experimental sessions can be performed without the need for recalibration, allowing for the exclusive use of colloidal probes. We use Eq. 1 to fit the retraction part of single indentation cycles performed with different colloidal probes (5, 6.62, and 10.2 *μ*m diameters) during independent experiments, imposing *γ* and *d* as free fit parameters. Droplets with sizes ranging from few hundreds of nanometers to few micrometers were used.

### FRAP-ID viscosity (*η*)

AFM-FS is employed with different AFM colloidal probe sizes (3.5, 5, and 10.2 *μ*m in diameter) exerting a high force (>10–20 nN) on regions wetted by droplets larger than 10 *μ*m, whose thickness ranged from 1 to 3 *μ*m (measured by AFM). Fast probe retract leads to local dewetting, associated with the formation of a micrometric hole (dry spot) within the condensed phase. Rewetting is monitored by epifluorescence microscopy until complete fluorescence recovery (Fig. 1 *c*). The closure of dry spots within liquid films has already been investigated in the literature at the macroscale (millimetric regime) (51,52). A lubrication model describes the fluid flow in terms of variation of the liquid depth (*h*) over time (*t*) in function of *γ*, liquid density, and gravity acceleration (51,52,54). We specifically address the boundary condition at the edges of the dry spot, relating the speed of the contact line velocity δa/δt to the static and dynamic contact angles (*θ* and δh/δr, respectively), using Tanner’s law (51,55,56,57), as shown by

(2) $$\frac{\delta a}{\delta t}=\frac{\gamma \omega }{2\eta }\left[\theta^{3}-{\left(\frac{\delta h}{\delta r}\right)}^{3}\right]\text{,}$$

where *a* is the radius of the dry spot, *ω* stands for the constant mobility of the contact line during hole collapse, and *h* represents the profile of the fluid depth, with δh/δr its derivative at the dry spot edges, which can be considered as the dynamic contact angle (Fig. 1 *c*). In this study we do not numerically solve the equation described in the lubrication model. Instead, we experimentally characterize δh/δr through epifluorescence frames acquired during rewetting (Fig. 1 *c*). To achieve this, fluorescence intensity is converted to fluid depth/thickness (*h*) upon calibration (for detailed protocol, see supporting material) and used to infer both δh/δr through numerical differentiation and the dry spot radius *a* using a threshold set to 10% of the initial *h*. Additionally, we track the larger hole radius *A*, determined with a threshold set to 90% of the initial *h*, to monitor complete fluorescence recovery, which requires longer times than substrate rewetting. Therefore, we distinguish two regimes in our dataset. 1) Regime I is the focus of FRAP-ID. It is characterized by a nearly constant δh/δr, resulting in the decrease of a(t) until hole collapse and complete rewetting (*a* = 0). Equation 2 holds in regime I, providing a reliable value of *η*, upon prior determination of *θ* and *γ*, in addition to the evaluation of the averaged δh/δr across the regime. To achieve this, we normalize Eq. 2 by considering only the time of the experiment (defined by the critical time $t_{c}$), hence imposing *ω* = 1. 2) Regime II is characterized by the recovery of the initial fluorescence achieved through the relaxation of the perturbed fluid morphology, leading to *A* = 0 (Fig. 1 *c*, *bottom*). Assuming the vertical fluid perturbation to be smaller than its characteristic in-plane length scale, the lubrication (thin film) model describing regime II relaxation is well described by (58,59):

(3) $$\partial_{t}h+\frac{\gamma }{3\eta }\nabla \cdot \left[h^{3}\nabla \left(\nabla^{2}h\right)\right]=0\text{.}$$

Given the circular spot within the fluid, Eq. 3 is invariant with the vertical axis and can be solved in an axisymmetric geometry, upon non-dimensionalization through $H=h/h_{i},R=r/h_{i}$, and $T=\frac{\gamma t}{3\eta h_{i}}$, where $h_{i}$ is the initial fluid depth, obtaining

(4) $$\partial_{T}H+\frac{1}{R}\partial_{R}\left[RH^{3}\left(\partial_{R}^{3}H+\frac{1}{R}\partial_{R}^{2}H-\frac{1}{R^{2}}\partial_{R}H\right)\right]=0\text{.}$$

Numerical simulations, conducted using a Runge-Kutta two-steps method, employ an experimental h(r) profile from regime II as initial condition. Mass conservation is imposed as a boundary condition while the fixed parameter η/γ is set from FRAP-ID and AFM-FS experiments. Comparison with regime II profiles is then carried out over the experimental time *t*.

## Results

Liquid droplet formation of the ELF3 protein is induced by a decrease in pH and dilution to prevent collisions with the AFM probe (44) (see supporting material for the detailed protocol). The static contact angle is evaluated from the AFM topography of several condensates (Fig. 2 *a*), acquired using a sharp AFM tip, and resulting in *θ* = 31° ± 10°. The latter was estimated using multiple morphological profiles across several droplets (reported as *light-blue segments* in Figs. 2 *a* and S1 *a*), leading to a contact angle population reported in Fig. S1 *b*. It is noteworthy that the Young-Laplace equation could estimate the surface tension from Fig. 2 *a* if the differential pressure (or density) between the condensed and dilute phases were known.

**Figure 3:**
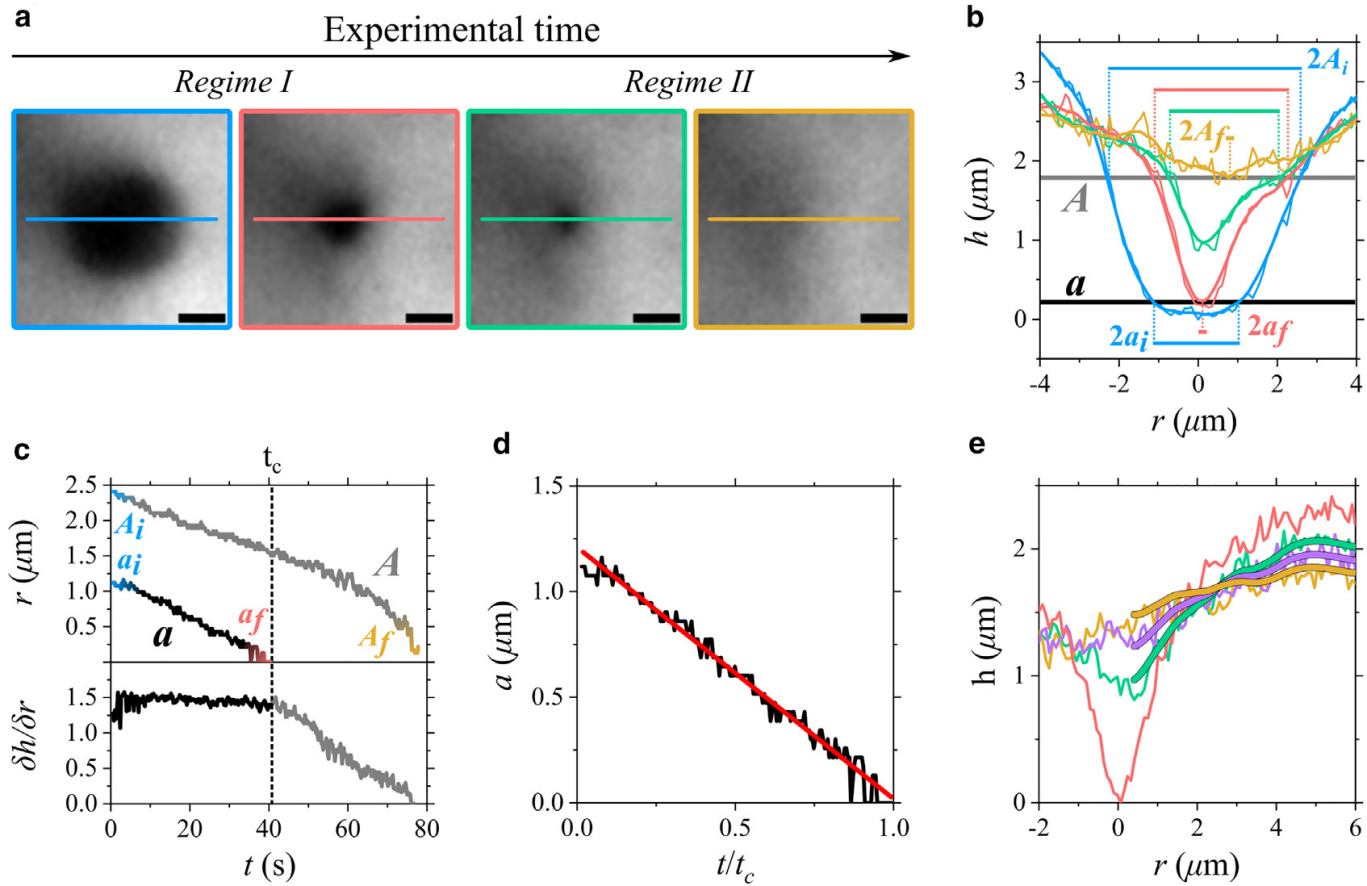
(*a*) Probe-induced dry spot exhibiting complete rewetting, from formation (*blue*) to closure (*yellow*) over time, observed through epifluorescence. Scale bars, 2 *μ*m. In each panel, the segment used to extract the geometric parameters is shown. (*b*) Hole geometrical profiles, color-coded as the segments in (*a*): the intercept with threshold values are used to determine the dry spot radius *a* (*black horizontal line*), the corresponding larger hole radius *A* (*gray horizontal line*), and the edge δh/δr. The intersection of such thresholds with the profiles defines the initial and final hole radii. Subscripts *i* and *f*, for *A* and *a*, denote the initial and final values, respectively. (*c*) Evolution of *A*, *a* (*top*), and δh/δr (*bottom*) during the time of the experiment. Complete rewetting occurs at *t* = $t_{c}$, when *a*≈ 0 (end of regime I, highlighted in boldface δh/δr). Regime II ($t>t_{c}$) is characterized by the recovery of the initial fluorescence through the relaxation of the fluid perturbation as tracked by *A* over time. (*d*) Evolution of hole radius *a* (*black*) as a function of the normalized time and associated best linear trend (*red*), providing an estimated viscosity. (*e*) Good agreement observed between numerical simulations (*thick lines*) and experimental profiles from regime II (*thin lines*) over a duration of 28 s.

Fig. 2*b* shows two indentation cycles performed onto two different droplets. The retract part and the associated best fit using Eq. 1 are shown in red and green, respectively. The surface tension distribution for the 5-*μ*m-diameter probe, made of borosilicate glass, is reported in the inset of Fig. 2 *b*, returning a value of 49 ± 9 *μ*N · m^−1^, corresponding to mean ± SD, and compatible with surface tension of other LLPS systems determined with coalescence experiments, OT, and other AFM experiments (2,15,17,41). The histograms related to tip diameters 6.62 and 10.2 *μ*m, made of silicon dioxide, are reported in Fig. S2, providing a surface tension in agreement with the data shown in Fig. 2 *b*, and suggesting that droplets similarly wet silicon dioxide and glass. The concurrent coalescence of droplets and the gradual wetting of the substrate, both observed through epifluorescence, alongside complete FRAP within a few minutes (Fig. S3), suggest that the droplets were in a liquid state throughout each AFM experimental session. However, the droplet mechanical response changes after ≈30 min post LLPS, resulting in indentation cycles exhibiting force steps during retraction, possibly due to intermolecular rupture events, which we interpret as due to transition to a gel state (44) (Fig. S1 *c*). Over time, droplets fuse and spread across the glass coverslip, resulting in the formation of larger condensates (films). Indentations at a high force induce dry spots followed by rewetting, whose timescale (tens of seconds) is reported in Fig. 3 and Video S1. The dry-spot profiles observed at different rewetting stages are plotted in Fig. 3 *b*. The corresponding dry spot radius (*a*), the larger radius (*A*), and δh/δr from each frame are plotted over time in Fig. 3 *c*. Upon determination of δh/δr during rewetting in regime I, the decrease of *a* with time leads to the evaluation of *η* over the time of the experiment $t_{c}$ (Fig. 3 *d*). The value of viscosity obtained (24 ± 14 Pa · s, corresponding to mean ± SD) is close to values reported by passive microrheology studies for other LLPS condensates (17). Complete fluorescence recovery is achieved within 1–3 min in regime II, tracked through the larger radius *A* and returning the initial fluid depth *h*. In this scenario, the observed timescale for fluorescence recovery is inferior to the timescale measured in FRAP experiments (Fig. S3 and supporting text). This discrepancy is potentially due to the fact that FRAP-ID data account for the entire mass flow, including confined populations that do not contribute to conventional FRAP experiments. Additional reasons might include the presence of multimers or particles of heterogeneous sizes and situations where labeling is not uniform, all of which can significantly impact FRAP data and, potentially, fluorescence correlation spectroscopy as well.

Video S1. AFM indentation inducing a dry spot within a thin fluorescent condensate Fluorescence recovery is due to rewetting, occurring on the timescale of tens of seconds.

Additionally, we observed several asymmetric closures due to the formation of dry spots with different δh/δr along the contact line: an averaged δh/δr was considered in these cases. If gravity and viscous forces are equilibrated within the fluid, the hole radius a(t) is proportional to ${\left(t_{c}-t\right)}^{\delta }$, with *δ* ≈ 1/10, as reported by Tanner in his first works (60). However, important deviations have been documented in the literature, and δ≈ 0.76 was observed by macroscopic studies (52,54). Our datasets indicate 0.7 < *δ* < 0.95 during rewetting. For δ= 0.95 (Fig. 3 *e*), δh/δr remains almost constant, and a nearly linear decrease of *a* with time is observed in regime I (Fig. 3 *c*). For lower *δ* values, we observed a gradual increase of δh/δr with time, leading us to estimate the viscosity through linear approximations of a(t) and δh/δr over a timescale shorter than the full time of the experiment. Comparison between fluid relaxation in regime II and numerical simulations carried out using Eq. 4 demonstrates a good agreement. An illustrative example is presented in Fig. 3 *e* over a period of 28 s, assuming an inverse capillary velocity of 0.49 s·*μ*m^−1^, as evaluated through FRAP-ID and AFM-FS. The initial h(r) (shown in *green*) is selected to ensure a vertical perturbation smaller than the in-plane perturbation length scale, a condition not met by the first profile of regime II (shown in *red*). Alternatively, one can iteratively adjust η/γ to achieve the best match between Eq. 4 numerical solutions and experiments. This approach allows for another evaluation of the η/γ parameter. Therefore, in summary, a single FRAP-ID measurement enables the assessment of η/γ (or directly *η* if *γ* is known from AFM-FS) through regime I, while regime II provides an independent re-evaluation of this parameter.

**Figure 4:**
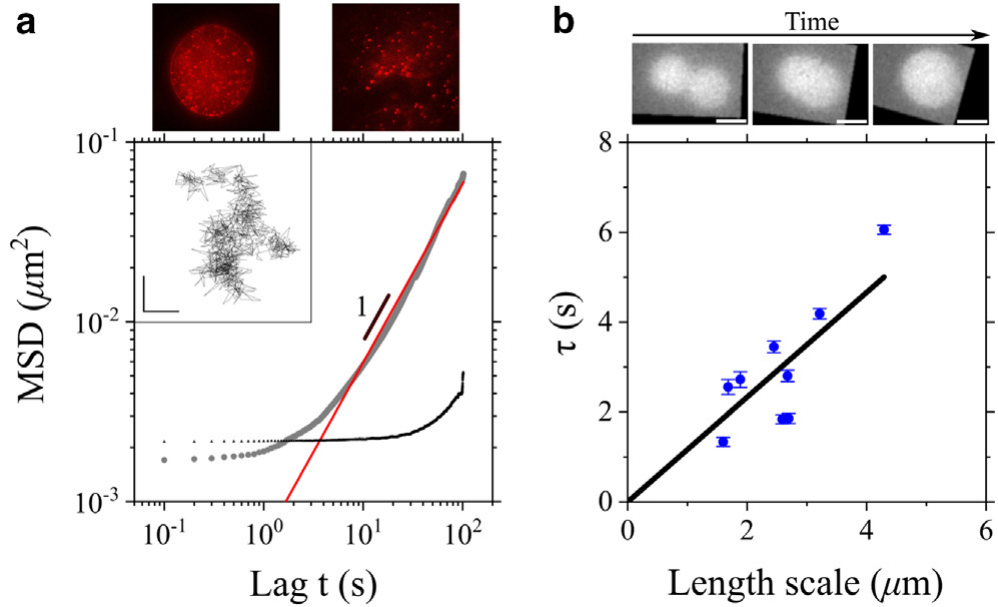
(*a*) Evaluation of droplet viscosity using passive microrheology. 50-nm (*left*) and 180-nm (*right*) beads embedded within protein condensates (*top*). The MSD vs. Lag *t* (*gray*) for 180-nm beads is fitted with a linear trend (*red*) to determine slope and intercept, the latter providing the diffusion coefficient. The instrumental background noise (*black*) is characterized by observing immobile particles in a separate experiment. The inset shows a particle trajectory. Scales bars on both *x* and *y* axis = 100 nm. (*b*) Coalescence between two droplets (*top*). Characteristic fusion time (*τ*) plotted against the droplet length scale (*blue circles*), and estimated linear trend (*black line*). Error bars derived from the exponential fit are shown.

To further validate our data, we compare the viscosity estimated through FRAP-ID with data from both microrheological experiments (Videos S2 and S3) and coalescence experiments (Video S4). Fig. 4 *a* reports a typical mean square displacement (MSD) as a function of lag time in a log-log plot, allowing the determination of the type of motion (*α* = 1 indicates Brownian motion), alongside the related fit that excludes the instrumental background noise (Fig. 4 *a* and supporting material). Considering curves with 0.9 <α< 1.1, the extrapolated diffusion coefficients lead to 34 Pa · s and 17 Pa · s for 50-nm- and 180-nm-diameter beads, respectively. Discrepancy may arise from variations in bead size and their interaction with the internal structures of the condensate, factors that can influence bead motion (36). Additionally, we observe a decrease of *α* with time, suggesting the rise of more confined trajectories. In agreement with the evolution of the force curves acquired by AFM, we ascribe this behavior to the liquid-to-gel state transition (droplet aging), partially accelerated by continuous exposure to the excitation laser used for epifluorescence imaging. Monitoring droplets coalescence across nine fusion events in separate experimental sessions provides the characteristic fusion time as a function of droplet length scale (Figs. 4 *b* and S5). The resulting inverse capillary velocity η/γ = 1.1 ± 0.2 s · *μ*m^−1^ is higher than the value obtained through AFM-FS and FRAP-ID, returning η/γ = 0.49 ± 0.30 s · *μ*m^−1^. The discrepancy can be attributed to several factors: 1) during coalescence, droplets are observed in solution, whereas in FRAP-ID they interact with the substrate; 2) coalescence experiments were conducted under slightly different protein concentration and salt conditions compared to those used for FRAP-ID. Such a difference was introduced to increase the probability to detect coalescence events within the camera’s field of view (see supporting material).

Video S2. Passive microrheology experiments employing 50-nm fluorescent beads diffusing within a condensate droplet

Video S3. Passive microrheology experiments Zoom of Video S2.

Video S4. Coalescence between two fluorescent condensate droplets

## Discussion

The sequential assessment of *γ* and *η* in a single experiment is a significant advantage, albeit subject to certain technical limitations. AFM-FS can estimate the surface tension of small droplets (<1 *μ*m) that cannot be characterized with other methods, down to a few *μ*N · m^−1^. It is suitable for viscous biomolecular condensates of variable size and nature/composition, exhibiting sufficient attractive forces (≥100 pN) to be measured by AFM. Indeed, optimization and increase of the AFM probe size might be necessary to enable the evaluation of a surface tension down to ≈1 *μ*N · m^−1^. FRAP-ID can be applied to dry spots with a size consistently larger than the diffraction limit, facilitating an accurate radius estimation. It is suited for condensates characterized by micrometric sizes, larger than the diameter of the tip used for dewetting, a condition which is usually reached after multiple coalescence events followed by wetting of the substrate. Dry spots collapsing within 4 s, observed through 21 frames at a rate of 5 frames per second, represent the fastest rewetting events we could properly analyze. Faster acquisition time can be beneficial only if fluorescence intensity signal-to-noise ratio is preserved. Therefore, for viscosities inferior to 1 Pa · s, the formation of larger dry spots is required, eventually using mechanical methods alternative to AFM. However, even in the absence of dry spot formation, low-viscosity condensates can be investigated using the lubrication analysis (regime II) introduced in this study. Regarding the accuracy of the FRAP-ID method, given the constant values for surface tension and contact angle used to estimate viscosity via Tanner’s law (Eq. 2), we attribute the resulting uncertainty primarily to the fluorescence intensity noise affecting the pixels of the acquisition camera (≈5%–10%). When propagated, this factor contributes significantly (15%–30%) to the statistical error reported for viscosity. Increasing the acquisition time for each fluorescence frame can effectively reduce the noise; however, this approach is feasible only for biomolecular condensates exhibiting slow dynamics.

Finally, it is worth noting the important deviation observed for *δ* in comparison with the expected value reported for Tanner’s law. In addition to cited cases (52,54), higher *δ* values have been reported for highly viscous fluids at early wetting stages (1/2 and 2/3). These observations are supported by experimental findings (61) and numerical simulations (62). Moreover, higher *δ* values have been reported for droplets spreading over rough surfaces and, importantly, for specific non-Newtonian fluids (*δ* = 1) (63). In the latter case, the formation of surface tension gradients is hypothesized to be caused by the presence of slow-moving molecules that cannot rapidly migrate to the newly created interface. The biomolecular condensates studied in this work possess a heterogeneous molecular composition. The presence of molecules exhibiting confined diffusion, in addition to static molecular populations, has already been observed in our previous work (44). Therefore, this heterogeneity can potentially lead to non-Newtonian fluid behavior, resulting in an increased *δ*. In perspective, comparison of our data with different LLPS systems can potentially provide new perspectives on the dynamics of biomolecular condensates, highlighting fluid mechanics via FRAP-ID as a candidate to evaluate the Newtonian nature of the liquid condensate phase.

## Conclusion

The methodology described in this paper offers the advantage of evaluating *γ* and *η* of biomolecular condensates in one single experiment, avoiding substrate functionalization and internalization of fluorescent beads, which is required for inverse capillary velocity measurements and passive microrheology, respectively. Moreover, this approach is particularly suitable for droplets with elevated surface tension in the mN · m^−1^ range, where AFM excels. Therefore, it offers a valuable alternative to observing coalescence events, which are often hindered by the rapidity of the process. We have shown that the force exerted by a droplet on a micrometric spherical probe can be used to characterize the surface tension in the tens of *μ*N · m^−1^ range, and the same probe can be used thereafter to locally induce the formation of a dry spot, whose subsequent rewetting can be monitored to extract droplet viscosity in the tens of Pa · s range. In this frame, rewetting is observed by tracking the fluorescence recovery due to the flow of GFP-tagged biomolecules within the condensed phase. A lubrication analysis of the subsequent fluid morphological relaxation can independently quantify the inverse capillary velocity, thereby cross-validating the values obtained from rewetting. This demonstrates the robustness of FRAP-ID. Furthermore, our findings are in good agreement with values obtained with passive microrheology, providing additional validation for this approach. Moreover, the viscosity assessed by FRAP-ID is a macroscopic property and hence independent of the presence of microdomains, which could influence the motion of fluorescent beads during microrheological experiments. The use of a simplified in vitro system to study and quantify LLPS parameters is an important first step in understanding the behavior of condensates and the physical characteristics of compartmentalized biological macromolecules. FRAP-ID is generally applicable to any in vitro LLPS-forming material, including synthetic polymers and complex mixtures of biological macromolecules. Indeed, our methodology can extend to broader applications, including nonbiological liquid-liquid interfaces. In these scenarios, surface tension and viscosity of micrometer-sized droplets vary depending on whether self-assembled materials decorate the interface. While quantifying these variations using conventional techniques at small micrometric scales may present challenges, FRAP-ID in combination with AFM-FS provides viable alternatives.

## Author contributions

A.S., C.M.D., C.Z., P.-E.M., and L.C. designed the research. A.S. and S.H. carried out protein purification. A.S. performed all experiments and analyzed the data. L.C. carried out simulations. All authors wrote the article.

## Supporting citations

References (64,65,66,67,68,69,70,71,72,73,74,75) appear in the supporting material.

## Supporting material

Document S1. Supporting text and Figures S1–S5

Document S2. Article plus supporting material
