# Supplementary material for: Quantifying surface tension and viscosity in biomolecular condensates by FRAP-ID
Source: Biophys J. 2024 Aug 8;123(19):3366–74. doi: 10.1016/j.bpj.2024.07.043 (PMC11480758; doi:10.1016/j.bpj.2024.07.043)
Supplement: Document S1. Supporting text and Figures S1–S5 [file mmc1.pdf]

Biophysical Journal, Volume *123*

Supplemental information

Quantifying surface tension and viscosity in biomolecular condensates by FRAP-ID

Andreas Santamaria, Stephanie Hutin, Christine M. Doucet, Chloe Zubieta, Pierre-Emmanuel Milhiet, and Luca Costa

Supplemental Material

MATERIALS

Bis-Tris propane (1,3-bis(tris(hydroxymethyl)methylamino)propane, purity ≥ 99%) was purchased from Merck; TCEP (Tris(2-carboxyethyl)phosphine hydrochloride, purity ≥ 98%) was purchased from Roth; sodium chloride (NaCl, purity ≥ 99%) was purchased from Euromedex; imidazole (purity ≥ 99%) and potassium chloride (KOH, purity ≥ 85%) were purchased from Merk; tris base (Tris[hydroxymethyl]aminomethane, purity ≥ 98.8%) was purchased from Merk Biorad; Ni Sepharose 6 Fast Flow Cytiva was purchased from Merk.

PROTEIN EXPRESSION AND PURIFICATION

ELF3 prion-like domain (Q7, residues 388-625, AT2G25930, *Arabidopsis thaliana* ecotype Columbia), tagged with Green Fluorescent Protein (GFP), was expressed in *Escherichia coli* BL21-CodonPlus-RIL cells (Agilent), as previously described (1, 2). Cells were resuspended in lysis buffer (100 mM Bis-Tris propane pH 9.4, 300 mM NaCl, 20 mM imidazole, 1 mM TCEP, plus 1x protease inhibitors (Roche)), lysed via sonication, spun at 45.000 x g to remove cell debris and the supernatant applied to a Ni-NTA column (1 mL of Ni Sepharose pre-equilibrated with resuspension buffer). The column was then washed with 50 column volumes (CV) of lysis buffer followed by a high salt wash (50 CV) with 100 mM Bis-Tris propane pH 9.4, 1 M NaCl, 20 mM imidazole, 1 mM TCEP. Finally, the proteins were eluted in 100 mM Bis-Tris propane pH 9.4, 300 mM NaCl, 300 mM imidazole, and 1 mM TCEP. Protein purity was determined by SDS-PAGE. The final protein concentration in the fraction used for all the experiments was 12.4 mg $\cdot$ mL^{-1}, calculated using A280 absorption with a NanoDrop instrument (NanoDrop one, ThermoFisher).

PROTEIN DROPLETS FORMATION

LLPS was triggered by a decrease in the pH of the solution. 4 μL of the protein solution in 100 mM Bis-Tris propane pH 9.4, 300 mM NaCl, 300 mM imidazole, and 1 mM TCEP were diluted with 4 μL of a buffering solution made up of 100 mM Tris buffer pH 7.5. This 1:1 mixing lead to a final pH of ≈ 8.2. The solution was deposited onto a glass coverslip (25 mm diameter, 0.165 mm thick, purchased from Marienfeld), previously cleaned with KOH 1M for 15 minutes in a sonication bath, and then extensively rinsed and sonicated for 15 minutes with MilliQ water. In such a crowded solution, collisions between large droplets and the AFM cantilever occur. Such collisions must be limited to ensure proper AFM data acquisition (1). Therefore, once the appearance of droplets in the solution deposited on the coverslip was assessed through fluorescence microscopy, 40 μL of a mixture of 33 mM Bis-Tris propane and 33 mM Tris buffer (pH=8.5) were added to dilute the solution to a final protein concentration of 1 mg $\cdot$ mL^{-1}, 42 mM NaCl, 36 mM Tris, 36 mM BTP and 0.08 mM TCEP, which drastically reduces droplets-AFM cantilever collisions. All our data acquisitions were conducted under these conditions, except for the coalescence experiments. In this specific case, we omitted the final dilution with 40 μL of the mixture containing 33 mM Bis-Tris propane and 33 mM Tris buffer (pH=8.5). This exclusion increased the probability of observing coalescence events within the camera's field of view.

CORRELATIVE ATOMIC FORCE MICROSCOPY - WIDE FIELD FLUORESCENCE MICROSCOPY

AFM coupled with a wide-field fluorescence microscope was developed in-house, allowing simultaneously AFM and fluorescence imaging (1, 3–8). It is based on a Nanowizard 4 (JPK Instruments, Bruker) mounted on a Zeiss inverted optical microscope, equipped with a LX 488-50 OBIS (488 nm, Coherent), a Sapphire 561-100 CW (561 nm, Coherent) and a F-04306-107 (642 nm, MPB) as laser sources and an oil immersion objective with a 1.46 numerical aperture (Plan-Apochromat 100X, Zeiss). Fluorescence was collected with an EmCCD iXon Ultra897 (Andor) camera. An ET800sp short-pass filter (Chroma) was used in the emission optical path to filter out the light source of the AFM optical beam deflection system. Epifluorescence images were acquired using the emission filter ET525/50 nm (Chroma) and an acousto-optic tunable filter (AOTFnc-400.650-TN, AA opto-electronics) to modulate the laser intensity and set the exposure time for each frame, typically between 100 and 400 ms. The setup includes a telescope to reach a final imaging magnification corresponding to a camera pixel size of 86 nm, resulting in fluorescence images 44.032 μm x 44.032 μm large. The 488 nm laser excitation power was measured before the objective with a PM100 energy meter (purchased from Thorlabs) and was optimized in all the experiments in the range of 0.3-1 μW.

- AFM imaging and contact angle evaluation

To determine the condensate contact angle after deposition on the glass coverslip, AFM images were acquired in Quantitative Imaging (QI) mode with a scan size of 50 μm × 50 μm and with 512 lines x 512 pixels. Images were acquired with a maximal force of 3 nN over oscillation cycles (Z length) of 2 μm. We used AC40TS AFM cantilevers, purchased from Olympus. The inverse optical lever sensitivity and the cantilever spring constant were calibrated using a combination of Sader (9) and thermal methods (10) in liquid environment (called *contact-free* method in the JPK AFM). Image treatment was performed using Gwyddion (11). Several morphological profiles were taken into account (see Fig. 2(a) and Fig. S1(a)) and a linear fit was performed to estimate the profile slope in correspondence to the edge of each wetting region, considered as the contact angle. The outcomes were averaged using a normal distribution, resulting in θ=31°±10° (Fig. S1(b)).

- AFM-Force Spectroscopy: surface tension

AFM cantilevers with colloidal probes with diameters equal to 5 μm (CP-CONT-BSG-A), 6.62 μm (CP-qp-CONT-SiO-C) and 10.2 μm (CP-PNPL-SiO-D), were purchased from NanoAndMore GmbH. The optical lever sensitivity was calibrated by performing indentation cycles on a rigid glass coverslip, while the spring constant was evaluated using the thermal method (10). AFM-Force Spectroscopy (AFM-FS) was performed through indentation cycles with a maximal force setpoint of 15 nN over probe-sample distances ranging from 4 to 8 μm (Z length), depending on the size of the droplet investigated. Only force curves in which both the approach and retract parts were flat at high tip-sample distance (D) were fitted. Among these, only the ones that returned a value of 0.05 μm $\leq d \leq$ 1 μm were taken into account for the final evaluation of δ, since Eq. 1 requires d to be much smaller than the probe radius. Moreover, spurious values of δ and also negligible secondary populations, probably due to condensate aging, were excluded (see Fig. S2). In particular, 85%, 81% and 92% of the total amount of the data were used for δ determination, for 5, 6.62 and 10.2 μm tip diameter, leading to 49 ± 9, 63 ± 23 and 37 ± 24 μN · m^{-1}, respectively. Surface tension data were fitted with a Gaussian distribution, and the associated results are reported as x_c±w, where x_c is the mean value and w is the standard deviation. This study involved the analysis of approximately 200 indentation curves acquired over six independent experimental sessions.

- Fluid depth/Fluorescence intensity calibration

AFM morphological images can be used for the fluid depth/fluorescence intensity calibration. Fluorescence and AFM topographical images are finely aligned and superimposed. For each pixel, the fluid height (depth) value was divided by the corresponding fluorescence intensity value, returning the calibration parameter of 0.25 nm·count^{-1} which is constant over the central region of the fluorescence image (30 μm x 30 μm). This value was employed to determine the droplet thickness h used in the FRAP-ID analysis.

- Viscosity: Fluorescence Recovery After Probe-Induced Dewetting (FRAP-ID)

In all FRAP-ID experiments, the laser power was set to 0.3 μW (488 nm). Dry spots were formed using colloidal AFM probes with diameters equal to 3.5 μm (CP-PNPL-SiO-B), 5 μm (CP-CONT-BSG-A) and 10.2 μm (CP-PNPL-SiO-D), purchased from NanoAndMore GmbH. Fluorescence images of probe-induced dry spots, saved in a stack of individual frames, are cropped so that the first frame corresponds to the initial dewetting, ensuring that the AFM probe had been placed far from the indentation region to avoid spurious fluorescence and probe reflections. Multiple profiles across the holes are extracted using ImageJ, exporting fluorescence intensity at each pixel for all time-dependent frames. Calibration for fluid depth/fluorescence intensity (0.25 nm·count^{-1}) and length/pixel ratio (86 nm·pixel^{-1}) are applied, obtaining fluid depth vs length profiles (Fig. 3(b)). The latter are low-passed filtered and the hole radii a and A are then evaluated using height thresholds, corresponding to 10% and 90% of the hole depth, respectively (depicted in grey and black in Fig. 3(b)). The experimental critical time (t_c), defined as the time necessary for the hole to collapse, corresponding to complete rewetting ($a \approx 0$), is here estimated from the fluorescence images, leading to ω = 1. Then, a is plotted over the experimental time (Fig. 3(c) top). Subsequently, $\delta h/\delta r$ is determined along the segment upon numerical differentiation at the edge of the hole, and is plotted vs time (Fig. 3(c) bottom). The latter is averaged over the rewetting time (t_c). Imposing θ and δ as fixed fit parameters, when $a(t)$ is linearly fitted using Eq. 2 (Fig. 3(d)) to assess the viscosity. In our experiments, we recorded an image each 200 or 400 ms. We analyzed fluorescence recovery data from 27 independent rewetting datasets, collected during four separate experimental sessions.

FLUORESCENCE RECOVERY AFTER PHOTOBLEACHING (FRAP)

Measurements were carried out with a correlative AFM-confocal microscope described in refs (1, 8) equipped with a supercontinuum laser (20 MHz, Rock-PP, Leukos) as laser source and an oil immersion objective with a 1.4 NA (Plan-Apochromat 100X, Zeiss). Fluorescence was collected after a 100 μm diameter pin-hole (P100D, Thorlabs) by an avalanche photodetector (SPCM-AQR-15, PerkinElmer) connected to an SPC-150 (Becker & Hickl) TCSPC card. The excitation laser was filtered with a ZET488/10 filter (Chroma) and the associated power was measured at the sample level with a S170C microscope slide power sensor and a PM100 energy meter (both purchased from Thorlabs). The power was set to 0.1 μW to image droplets, and to 150 μW to induce photobleaching (exposure time = 1 s). Confocal images and FRAP were acquired using a 525/39 nm (Thorlabs) emission filter. FRAP datasets exhibit complete fluorescence recovery within 5-10 minutes (Fig. S3), slower than the timescale observed in FRAP-ID rewetting experiments (30 s - 1 minute), although comparable with regime II timescale (fluid relaxation). FRAP data could not be properly fitted considering a single population, suggesting the presence of diffusing entities of different sizes and showing the complexity of the LLPS system investigated in this study.

PASSIVE MICRORHEOLOGY

Fluorescent spheres with diameters of 50 nm (40 nm nominal, 540/560 nm, purchased from Invitrogen F8792) and 180 nm (170 nm nominal, 633/660 nm, purchased from Life technologies P7220) were used to assess condensate viscosity through passive microrheology, in separate experiments. A solution of beads in water was mixed to the protein solution before pH drop and LLPS triggering. The same fluorescence microscope previously described was used to visualize 30 to 90 embedded beads moving within each condensate. Data were acquired at 10 frames $\cdot$ s^{-1} with laser power equal to 240 μW and 3 μW, for 561 nm and 642 nm laser sources, respectively. Data were treated with PaTrack software (12), which calculates the mean square displacement (MSD) of the beads as a function of lag time (Fig. S4).

$$MSD(n\delta t) = \frac{1}{N-n-1}\sum_{j=1}^{N-n-1}\{[x(j\delta t + n\delta t) - x(j\delta t)]^2 - [y(j\delta t + n\delta t) - y(j\delta t)]^2\} \tag{S1}$$

where δt is the time interval between two successive frames (here 100 ms), N is the total number of frames (here $\approx$ 1000, corresponding to 100 s), n is the number of time intervals, $x(t)$ and $y(t)$ are the bead coordinates at time t (13, 14). The MSD vs t profiles were fitted using an exponential model MSD= $4Dt^{\alpha}$, where D is the diffusion coefficient, and α indicates the type of motion ($\alpha < 1$, confined or subdiffusive; α=1 Brownian; $\alpha > 1$ superdiffusive). The initial $\approx$ 20% of the dataset is situated within the instrumental background noise and, consequently, was not taken into account. The background noise was determined by evaluating the MSD of immobile beads deposited onto a glass coverslip (Fig. 4(a)).

Data were displayed in a log-log plot, and only the curves with $0.9 \leq \alpha \leq 1.1$ were considered to determine condensate viscosity. The diffusion coefficient was determined from the intercept, leading to the viscosity of 43 Pa·s and 17 Pa·s for 50 and 180 nm beads, respectively. We imaged each condensate for $\approx$ 400 s. Data were then divided into ensembles of 100 s and treated separately, We observed a slow decrease of α with time, suggesting the appearance of more confined trajectories, which we ascribe to the aging process of the droplets. Additionally, to prevent interaction of the beads with ELF3 proteins in the condensed phase, we prepared PEGylated beads, as described in refs (15, 16). However, to the best of our observations, PEGylated beads did not embed within ELF3 droplets. This suggests that a different beads functionalization strategy is required, contributing to the complexity of data acquisition and interpretation in passive microrheology (17, 18), as mentioned in the main manuscript. A total of 642 trajectories were observed across four distinct experimental sessions.

COALESCENCE EXPERIMENTS

Fluorescence images, acquired at 10 frames·s^{-1}, were treated with ImageJ (19, 20). The first frame showing mechanical contact between the two droplets was considered as the starting of the fusion event (t=0). In case of mobile droplets, the StackReg plugin (21) was used to correct for the drift of the images. Subsequently, a mask was applied to individual droplets, and their elliptical shape was tracked over time (Fig. S5(a)). Then, the aspect ratio, A.R., the ratio of the two axis of the obtained ellipse), was plotted against time and fitted with an exponential function:

$$A.R. = 1 + ke^{-\frac{t}{\tau}} \tag{S2}$$

where k=A.R.$_{(t=0)} - 1$. The characteristic fusion time (τ) was plotted against the average droplet length scale, defined as $\sqrt{(major\ axis_{t=0} - minor\ axis_{t=0}) \cdot minor\ axis_{t=0}}$ (Fig. S5(b)). Finally, data were fitted with a linear curve imposing the

intercept equal to 0, obtaining the slope (inverse capillary velocity) $\eta/\gamma = 1.1 \pm 0.2$ s$\cdot\mu$m^{-1}. We monitored nine coalescence events, collected during two experimental sessions.

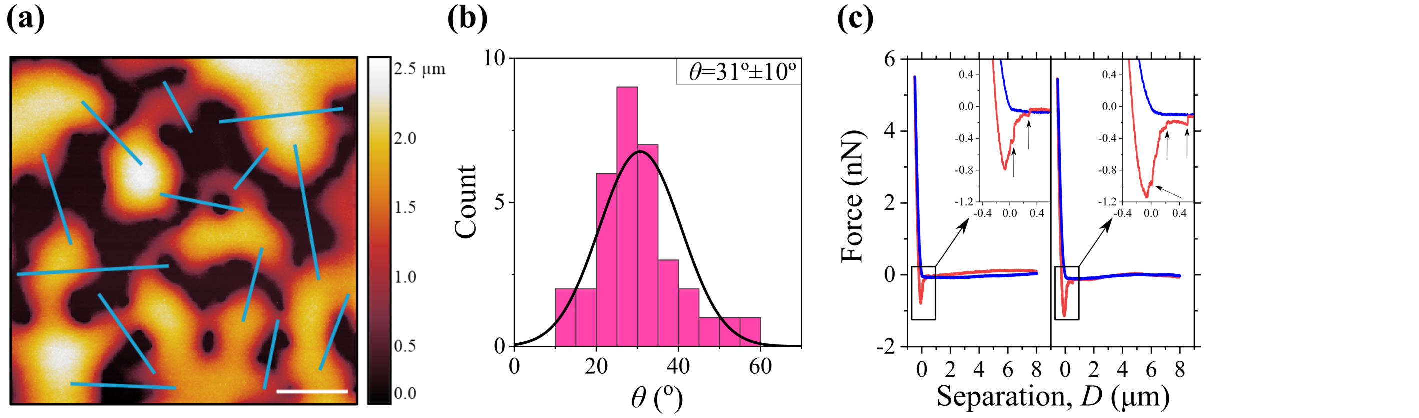

Figure S1: **(a)** AFM image (512 x 512 pixels) of droplets wetting large regions of a glass coverslip. Scale bar (white segment) = 10 μm. The profiles depicted in light blue (thickness 5 pixels) were taken into account to extract the contact angle θ. **(b)** Normal distribution of all the values obtained for θ. **(c)** Example of two indentation cycles on an aged (gel) droplet, exhibiting several force steps (arrows) while retracting the probe from the droplet and suggesting intermolecular ruptures. The blue curve is the approach, the red is the retract.

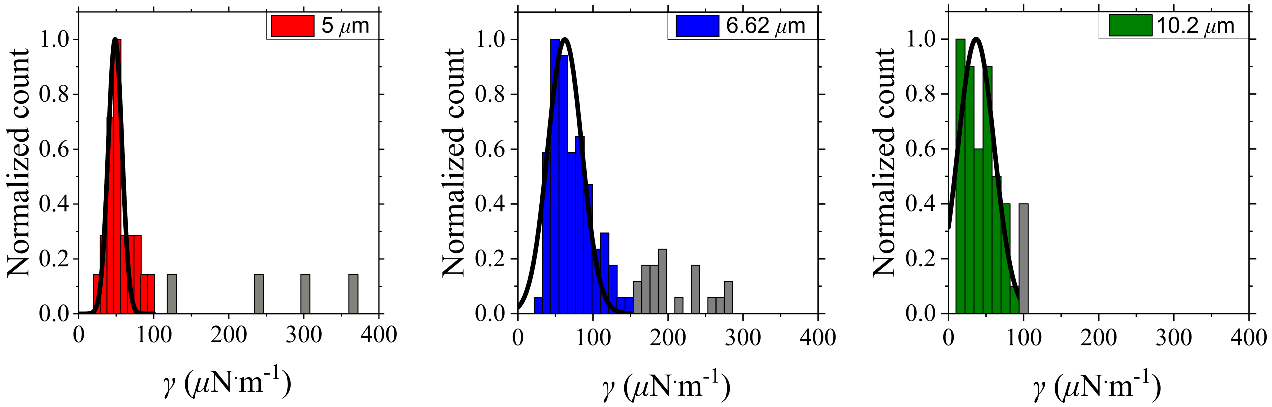

Figure S2: Surface tension distributions obtained with different AFM colloidal probe sizes. The black lines represent the associated Gaussian best fit distribution. Negligible secondary populations, probably due to condensate aging, were excluded from the fit (grey).

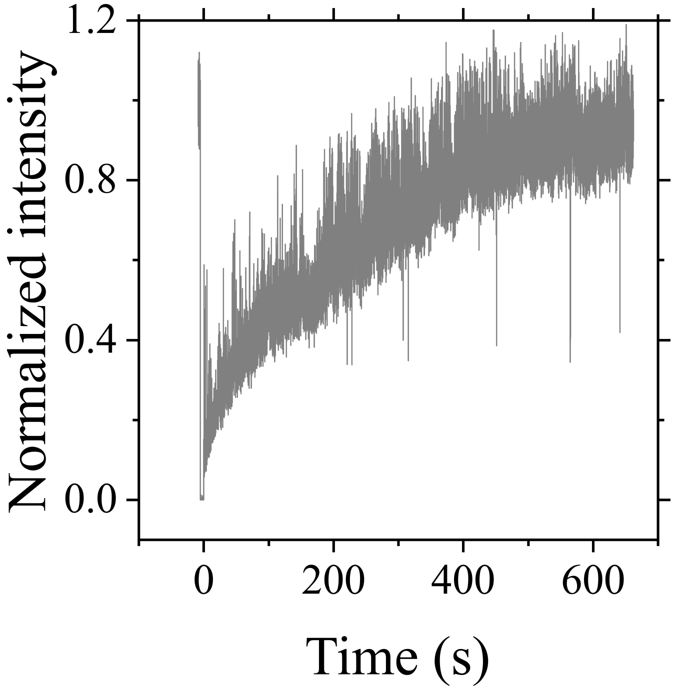

Figure S3: Recurrent FRAP dataset, showing complete recovery within a few minutes.

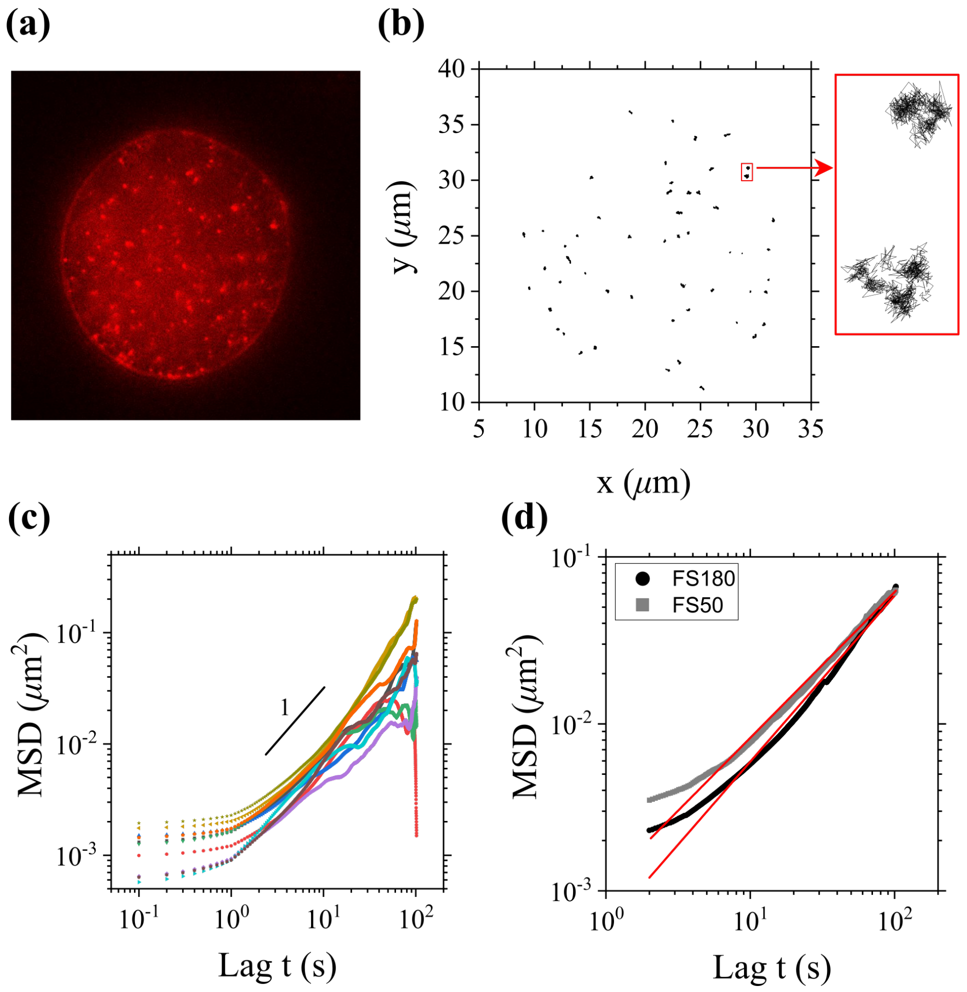

Figure S4: **(a)** 50 nm beads embedded into a droplet. Panel **(b)** shows several beads trajectories over 100 s, alongside with a zoom of two of these. **(c)** Examples of typical MSD curves for single 50 nm beads embedded within a condensate. **(d)** Averaged MSD curves for 50 nm and 180 nm fluorescent beads embedded in one liquid droplet, with associated fit.

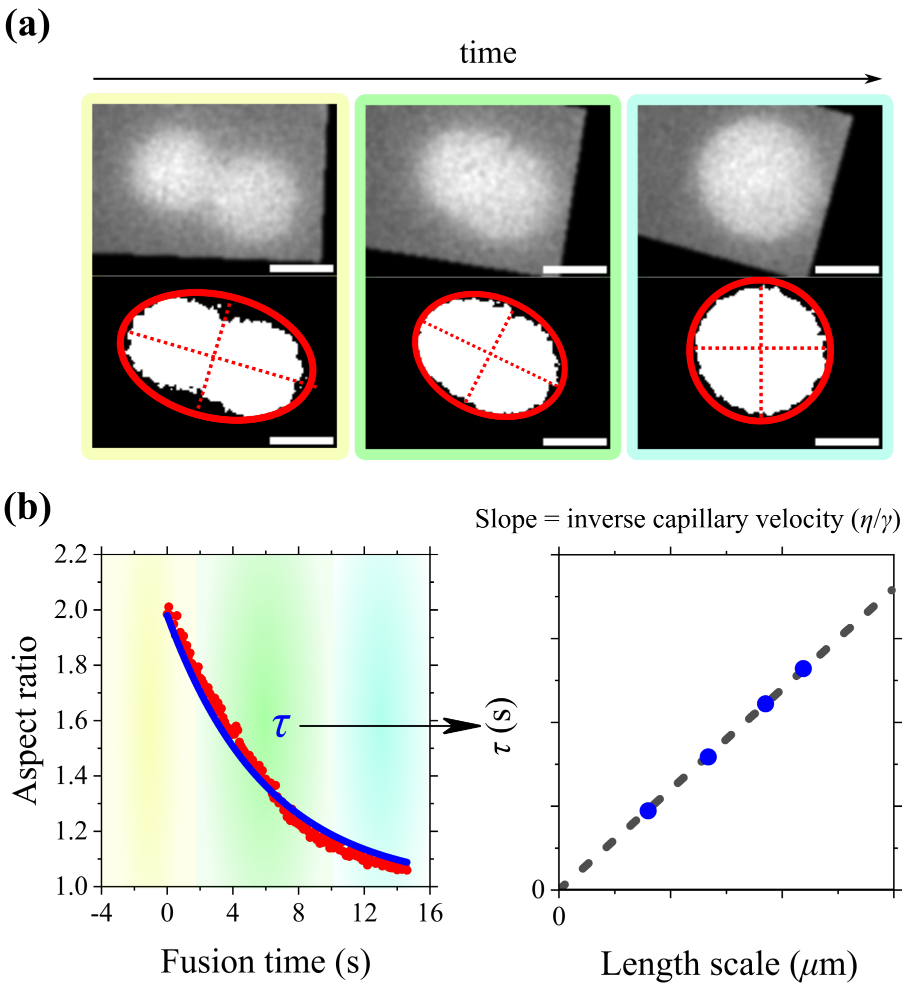

Figure S5: **(a)** Images reporting coalescence of two droplets, from first contact, t=0 (yellow panel), until complete fusion (cyan panel). Scale bars = 3 μm. The bottom panels show the mask applied to follow the fusion events, including the red ellipse and circle indicating the output of droplet segmentation (*Analyze particles* tool in ImageJ). The red dotted lines indicate the major and minor axis, used to calculate the aspect ratio. The data treatment was performed following the example of ref (22). **(b)** The left panel reports the aspect ratio plotted against fusion time (red circles) and the corresponding exponential fit (blue line). From the fit, the characteristic fusion time (τ) is determined. The right panel shows an ideal representation of the evaluation of the inverse capillary velocity (real data in Fig. 4 in main manuscript).

SUPPORTING REFERENCES

1. Hutin, S., J. R. Kumita, V. I. Strotmann, A. Dolata, W. L. Ling, N. Louafi, A. Popov, P.-E. Milhiet, M. Blackledge, M. H. Nanao, P. A. Wigge, Y. Stahl, L. Costa, M. D. Tully, and C. Zubieta, 2023. Phase separation and molecular ordering of the prion-like domain of the Arabidopsis thermosensory protein EARLY FLOWERING 3. Proc. National Acad. Sci. 120:e2304714120. https://www.pnas.org/doi/abs/10.1073/pnas.2304714120.

2. Jung, J. H., A. D. Barbosa, S. Hutin, J. R. Kumita, M. Gao, D. Derwort, C. S. Silva, X. Lai, E. Pierre, F. Geng, S. B. Kim, S. Baek, C. Zubieta, K. E. Jaeger, and P. A. Wigge, 2020. A prion-like domain in ELF3 functions as a thermosensor in Arabidopsis. Nature 585:256–260. https://doi.org/10.1038/s41586-020-2644-7.

3. Vial, A., C. Taveneau, L. Costa, B. Chauvin, H. Nasrallah, C. Godefroy, P. Dosset, H. Isambert, K. X. Ngo, S. Mangenot, D. Levy, A. Bertin, and P. E. Milhiet, 2021. Correlative AFM and fluorescence imaging demonstrate nanoscale membrane remodeling and ring-like and tubular structure formation by septins. Nanoscale 13:12484–12493. https://doi.org/10.1039/D1NR01978C.

4. Dahmane, S., C. Doucet, A. Le Gall, C. Chamontin, P. Dosset, F. Murcy, L. Fernandez, D. Salas, E. Rubinstein, M. Mougel, M. Nollmann, and P. E. Milhiet, 2019. Nanoscale organization of tetraspanins during HIV-1 budding by correlative dSTORM/AFM. Nanoscale 11:6036–6044. https://pubs.rsc.org/en/content/articlehtml/2019/nr/c8nr07269h.

5. Vial, A., L. Costa, P. Dosset, P. Rosso, G. Boutières, O. Faklaris, H. Haschke, P. E. Milhiet, and C. M. Doucet, 2023. Structure and mechanics of the human nuclear pore complex basket using correlative AFM-fluorescence superresolution microscopy. Nanoscale 15:5756–5770. https://doi.org/10.1039/D2NR06034E.

6. Elena-Real, C. A., A. Sagar, A. Urbanek, M. Popovic, A. Morató, A. Estaña, A. Fournet, C. Doucet, X. L. Lund, Z. D. Shi, L. Costa, A. Thureau, F. Allemand, R. E. Swenson, P. E. Milhiet, R. Crehuet, A. Barducci, J. Cortés, D. Sinnaeve, N. Sibille, and P. Bernadó, 2023. The structure of pathogenic huntingtin exon 1 defines the bases of its aggregation propensity. Nat. Struct. Mol. Biol. 2023 30:3 30:309–320. https://www.nature.com/articles/s41594-023-00920-0.

7. Schatz, M., L. Marty, C. Ounadjela, P. B. V. Tong, I. Cardace, C. Mettling, P.-E. Milhiet, L. Costa, C. Godefroy, M. Pugnière, J.-F. Guichou, J.-M. Mesnard, M. Blaise, and B. Beaumelle, 2023. A Tripartite Complex HIV-1 Tat-Cyclophilin A-Capsid Protein Enables Tat Encapsidation That Is Required for HIV-1 Infectivity. J. Virol. 97:e00278–23. https://doi.org/10.1128/jvi.00278-23.

8. Fernandes, T. F. D., O. Saavedra-Villanueva, E. Margeat, P.-E. Milhiet, and L. Costa, 2020. Synchronous, Crosstalk-free Correlative AFM and Confocal Microscopies/Spectroscopies. Sci. Reports 10:7098. https://doi.org/10.1038/s41598-020-62529-3.

9. Sader, J. E., R. Borgani, C. T. Gibson, D. B. Haviland, M. J. Higgins, J. I. Kilpatrick, J. Lu, P. Mulvaney, C. J. Shearer, A. D. Slattery, P. A. Thorén, J. Tran, H. Zhang, H. Zhang, and T. Zheng, 2016. A virtual instrument to standardise the calibration of atomic force microscope cantilevers. Review Sci. Instruments 87. https://doi.org/10.1063/1.4962866.

10. Proksch, R., T. E. Schäffer, J. P. Cleveland, R. C. Callahan, and M. B. Viani, 2004. Finite optical spot size and position corrections in thermal spring constant calibration. Nanotechnology 15:1344–1350. https://iopscience.iop.org/article/10.1088/0957-4484/15/9/039.

11. Nečas, D., and P. Klapetek, 2012. Gwyddion: An open-source software for SPM data analysis. Central Eur. J. Phys. 10:181–188. https://doi.org/10.2478/s11534-011-0096-2.

12. Dosset, P., P. Rassam, L. Fernandez, C. Espenel, E. Rubinstein, E. Margeat, and P. E. Milhiet, 2016. Automatic detection of diffusion modes within biological membranes using back-propagation neural network. BMC Bioinform. 17:1–12. http://dx.doi.org/10.1186/s12859-016-1064-z.

13. Qian, H., M. P. Sheetz, and E. L. Elson, 1991. Single particle tracking. Analysis of diffusion and flow in two-dimensional systems. Biophys. J. 60:910–921. http://dx.doi.org/10.1016/S0006-3495(91)82125-7.

14. Kusumi, A., Y. Sako, and M. Yamamoto, 1993. Confined lateral diffusion of membrane receptors as studied by single particle tracking (nanovid microscopy). Effects of calcium-induced differentiation in cultured epithelial cells. Biophys. J. 65:2021–2040. http://dx.doi.org/10.1016/S0006-3495(93)81253-0.

15. Garting, T., and A. Stradner, 2019. Synthesis and application of PEGylated tracer particles for measuring protein solution viscosities using Dynamic Light Scattering-based microrheology. Colloids Surfaces B: Biointerfaces 181:516–523. https://doi.org/10.1016/j.colsurfb.2019.05.059.

16. Daniels, B. R., B. C. Masi, and D. Wirtz, 2006. Probing single-cell micromechanics in vivo: The microrheology of C. elegans developing embryos. Biophys. J. 90:4712–4719. https://doi.org/10.1529/biophysj.105.080606.

17. Alshareedah, I., K. Taranpreet, and P. R. Banerjee, 2021. Methods for Characterizing the Material Properties of Biomolecular Condensates. Methods Enzymol. 646:143–183. https://doi.org/10.1016/bs.mie.2020.06.009.

18. Feric, M., and C. P. Brangwynne, 2013. A nuclear F-actin scaffold stabilizes RNP droplets against gravity in large cells. Nat. Cell Biol. 2012 14:5 15:1253–1259. https://doi.org/10.1038/ncb2830.

19. Schneider, C. A., W. S. Rasband, and K. W. Eliceiri, 2012. NIH Image to ImageJ: 25 years of image analysis. Nat. Methods 2012 9:7 9:671–675. https://www.nature.com/articles/nmeth.2089.

20. Abràmoff, M. D., P. J. Magalhães, and S. J. Ram, 2004. Image processing with imageJ. Biophotonics Int. 11:36–41.

21. Thévenaz, P., U. E. Ruttimann, and M. Unser, 1998. A pyramid approach to subpixel registration based on intensity. IEEE Trans. on Image Process. 7:27–41. https://doi.org/10.1109/83.650848.

22. Ceballos, A. V., C. J. McDonald, and S. Elbaum-Garfinkle, 2018. Methods and Strategies to Quantify Phase Separation of Disordered Proteins. Methods Enzymol. 611:31–50. https://doi.org/10.1016/bs.mie.2018.09.037.
