# Supplementary material for: Quantifying surface tension and viscosity in biomolecular condensates by FRAP-ID
Source: Biophys J. 2024 Aug 8;123(19):3366–74. doi: 10.1016/j.bpj.2024.07.043 (PMC11480758; doi:10.1016/j.bpj.2024.07.043)
Supplement: Document S2. Article plus supporting material [file mmc6.pdf]

Biophysical Journal
Article

Biophysical Society

Quantifying surface tension and viscosity in biomolecular condensates by FRAP-ID

Andreas Santamaria,[1] Stephanie Hutin,[2] Christine M. Doucet,[1] Chloe Zubieta,[2] Pierre-Emmanuel Milhiet,[1] and Luca Costa[1,*]
[1]Center for Structural Biology (CBS), CNRS, INSERM, Montpellier University, Montpellier, France and [2]Laboratoire de Physiologie Cellulaire et Végétale, Université Grenoble-Alpes, CNRS, CEA, INRAE, IRIG-DBSCI, Grenoble, France

ABSTRACT Many proteins with intrinsically disordered regions undergo liquid-liquid phase separation under specific conditions in vitro and in vivo. These complex biopolymers form a metastable phase with distinct mechanical properties defining the timescale of their biological functions. However, determining these properties is nontrivial, even in vitro, and often requires multiple techniques. Here we report the measurement of both viscosity and surface tension of biomolecular condensates via correlative fluorescence microscopy and atomic force microscopy (AFM) in a single experiment (fluorescence recovery after probe-induced dewetting, FRAP-ID). Upon surface tension evaluation via regular AFM-force spectroscopy, controlled AFM indentations induce dry spots in fluorescent condensates on a glass coverslip. The subsequent rewetting exhibits a contact line velocity that is used to quantify the condensed-phase viscosity. Therefore, in contrast with fluorescence recovery after photobleaching (FRAP), where molecular diffusion is observed, in FRAP-ID fluorescence recovery is obtained through fluid rewetting and the subsequent morphological relaxation. We show that the latter can be used to cross-validate viscosity values determined during the rewetting regime. Making use of fluid mechanics, FRAP-ID is a valuable tool to evaluate the mechanical properties that govern the dynamics of biomolecular condensates and determine how these properties impact the temporal aspects of condensate functionality.

SIGNIFICANCE Biomolecular condensates, resulting from the liquid-liquid phase separation of proteins, exhibit distinct surface tension and viscosity defining the timescale of their biological functions. Making use of fluid mechanics, these parameters are determined using a method that we named fluorescence recovery after probe-induced dewetting, FRAP-ID. Upon deposition onto a glass coverslip, fluorescent condensates are indented with a micrometric probe, leading to the formation of dry spots. The subsequent fluorescence recovery, characterized by fluid rewetting and morphological relaxation, is used to evaluate viscosity and surface tension.

INTRODUCTION

Biomolecular condensates, formed by liquid-liquid phase separation (LLPS) of macromolecules (1–3) such as proteins, DNA, lipids, and glycogen, are essential for subcellular compartmentalization via the formation of membrane-less organelles (4). Examples of biomolecular condensates include the nucleolus and actin-mediated structures (5–7). These structures are believed to play an essential role by increasing local concentration of the partitioning molecules (4). Additionally, dysfunctions related to LLPS formation, dynamics, and associated mechanical properties, due to transitions to a solid/gel state for instance, are often related to the onset of pathologies (4,8–14). In this framework, properties such as viscosity (η) and surface tension (γ) between dilute and condensed phases, which govern LLPS structure and mechanics (15,16), are key parameters that need to be quantified in order to investigate condensate dynamics (e.g., droplet fusion), function, aging, molecular recruitment, diffusive processes, and condensate internal organization (17–22). Fluorescence recovery after photobleaching (FRAP) is the technique widely used to gain insights into biomolecular condensate fluidity, qualitatively estimating the diffusion coefficient of the condensed-phase component in vitro and in vivo (6,9,18,23–31). However, interpreting FRAP results (25,30) can be complex, especially for multicomponent condensates whose different size or nature results in

Submitted April 8, 2024, and accepted for publication July 31, 2024.
*Correspondence: luca.costa@cnrs.fr
Editor: Sudipta Maiti.
https://doi.org/10.1016/j.bpj.2024.07.043
© 2024 The Author(s). Published by Elsevier Inc. on behalf of Biophysical Society.
This is an open access article under the CC BY license (http://creativecommons.org/licenses/by/4.0/).

multiple recovery velocities or static behavior. Passive microrheology is another technique used to determine the viscosity of the condensates and employs fluorescence microscopy to track the movement of fluorescent beads (usually ranging from 20 to 500 nm in diameter and functionalized with polyethylene glycols (30)), embedded within condensates (17,30–35). Applying the Stokes-Einstein equation allows for the evaluation of reliable viscosity values, provided that beads, located at a considerable distance from the edges of the droplets, undergo Brownian motion, therefore requiring the preparation of large droplets, which may be challenging even in vitro (17). Factors such as functionalization of the beads, their size, and the presence of heterogeneous micro- and nanodomains introduce additional parameters that contribute to the complexity of data interpretation (30,36). Active microrheology provides an attractive and more versatile alternative to these techniques via the use of optically trapped beads to measure condensate viscosity (37). Previous studies have demonstrated that atomic force microscopy (AFM) may be employed to investigate polymeric and biomolecular condensates in the liquid phase (15,38,39) or, more generally, liquid droplets and particles (40). Rheological properties can be obtained upon droplet confinement between the substrate and a micrometric spherical (colloidal) AFM probe, assuming that the system is at equilibrium at small contact angles. This approach reveals, unsurprisingly, consistently different viscoelastic values across the frequency spectrum (15). In contrast, conventional AFM quasi-static indentation cycles provide values under stationary conditions, as demonstrated in (39). The second important parameter to be determined is the surface tension of the sample. This can be assessed with optical tweezers (OT) (41), which trap fluorescent particles within droplets and subject them to an oscillatory trajectory. Monitoring the condensate response enables the evaluation of both surface tension and rheological behavior. However, optical traps are not suitable for studying droplets formed by thermoresponsive biomolecules, due to the high laser power employed, nor for condensates with high surface tension, as OT measurements are restricted to piconewton-range forces (15).

The inverse capillary velocity, defined by the ratio η/γ, can be determined by monitoring the coalescence between two droplets (2,5,12,17,18,30,32,42). The characteristic fusion time (τ) is determined by monitoring the evolution of an elliptic area encompassing two droplets into a circular area with a single droplet, the final stage of the fusion event. The inverse capillary velocity acts as proportionality constant between τ and the droplet length scale, enabling the evaluation of γ, after previous determination of η by microrheology. Mainly used for micrometric and optically visible droplets, these experiments may require surface functionalization or the use of OT to avoid wetting or induce droplet fusion (24,28,29,31). Additionally, the determination of the elliptic area can be technically challenging (30), particularly in scenarios involving high surface tension in the range of millinewtons per meter or low viscosity, where accurately characterizing the rapid fusion process becomes difficult. In this manuscript, we propose a straightforward method that employs correlative and simultaneous atomic force and fluorescence microscopies (43–49) to determine γ and η of fluorescently labeled biomolecular condensates in a single experiment in vitro. Initially, the condensate is confined between a colloidal AFM probe and the substrate (see experimental setup shown in Fig. 1 *a*), whose mutual adhesive force allows us to determine the surface tension of the condensate (50) (Fig. 1 *b*). Small droplets, with size inferior to the diffraction limit, can be characterized in this way, in contrast to the techniques previously described that require large droplets. Subsequently, in a scenario where multiple droplets have wetted the substrate, covering large micrometric regions, dry spots are formed within these areas by applying an AFM force higher than 10–20 nN. Recording the fluorescent mass flow during rewetting until hole collapse (Fig. 1 *c*) enables the evaluation of the condensate viscosity (51,52). We name this technique FRAP-ID (fluorescence recovery after probe-induced dewetting). As a case of study, we use the prion-like domain of the EARLY FLOWERING 3 (ELF3) protein, tagged with Green Fluorescent Protein (GFP), undergoing LLPS. The unstructured prion-like domain present in ELF3 is responsible for driving condensate formation, in vitro and in vivo, in a temperature- and pH-dependent manner (44,53), and provides a robust and easily manipulated model system for these studies. Our results are then compared with data generated using passive microrheology, coalescence monitoring, and FRAP. The comparison reveals strong agreement with the obtained results. The detailed analysis of our methodology reveals the capability to evaluate surface tension as low as a few micronewtons per meter and viscosity down to 1 Pa · s. Further improvements are achievable by tuning the cantilever spring constant and adjusting the AFM probe size.

MATERIALS AND METHODS

Surface tension (γ)

By means of epifluorescence, single droplets can be aligned below the AFM probe. Using AFM-force spectroscopy (AFM-FS) with moderate force (<10–20 nN), droplets can be sequentially confined between the AFM probe and the glass coverslip, acting as a concave meniscus that leads to the adhesive attraction of the solid surfaces (Fig. 1 *b*). Such attractive force is directly proportional to γ, and force-versus-distance indentation cycles exhibit a hysteretic behavior (38). In the case of a colloidal probe of radius R, the attractive force measured while retracting the probe can be expressed as follows (50):

$$F(D) = -\frac{4\pi R\gamma\cos\theta}{1+\dfrac{D}{d}}, \tag{1}$$

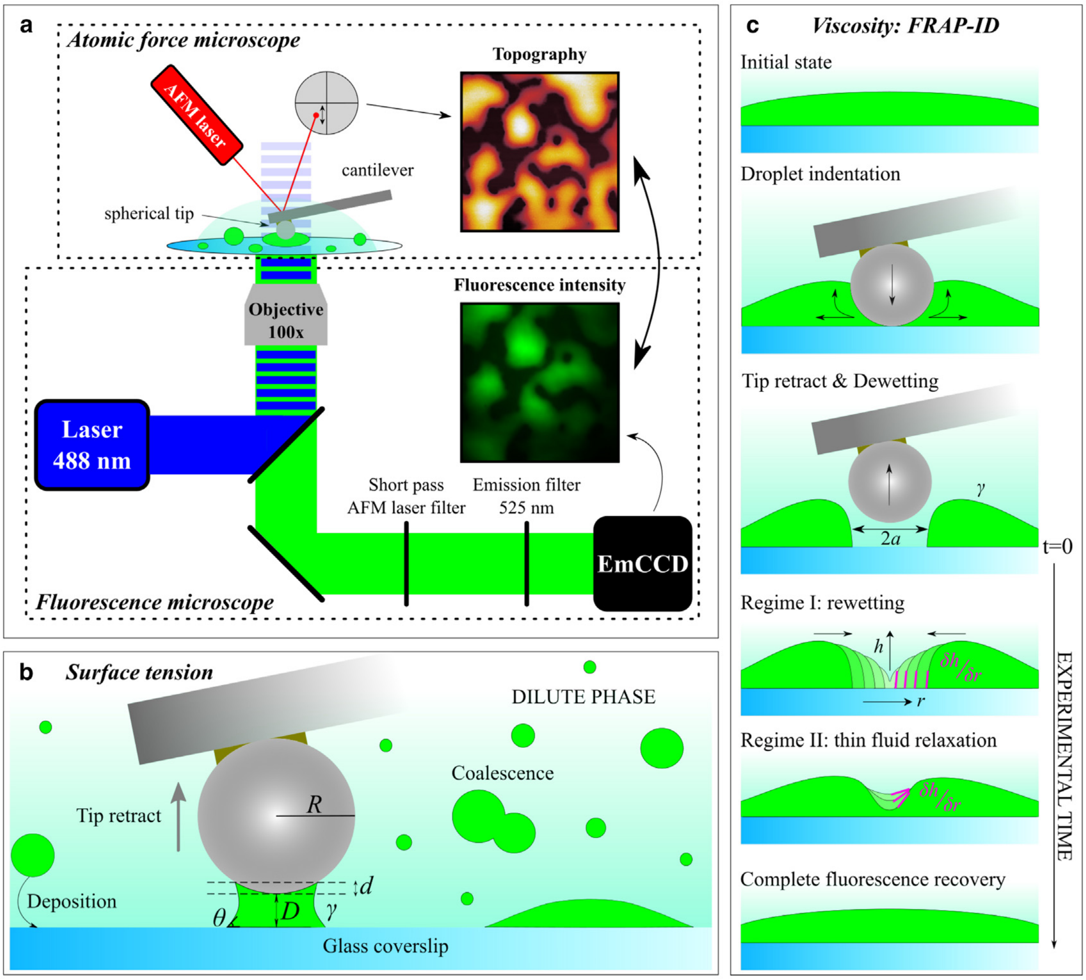

FIGURE 1 (*a*) Correlative AFM-epifluorescence microscopy setup. (*b*) Pictorial representation of a liquid droplet confined between the AFM colloidal probe with radius R and the glass substrate, and exerting an attractive force on the retracting probe. The force is used to assess the condensate surface tension (γ). (*c*) Sketch reporting the different phases of the FRAP-ID method. The formation of a dry spot (*top*) within the condensed phase (*green*) is tracked until complete rewetting (regime I). The contact line speed during this process is monitored through epifluorescence to determine the viscosity (η). Subsequently, complete fluorescence recovery is obtained through a fluid relaxation, whose lubrication analysis leads once again to η/γ, cross-validating values obtained from regime I.

where θ is the static contact angle at the three-phase boundary (substrate-condensed phase-dilute phase), D is the probe-substrate distance, and d is the height of the spherical probe cap that is wetted by the condensed phase. Eq. 1 holds for $R \gg d$ and therefore is applicable for cases in which droplets are much smaller than the colloidal probe. Fig. 1 *b* shows these parameters, alongside a schematic representing a droplet of the condensed phase (depicted in *bright green*) confined between AFM probe and substrate, immersed in the dilute phase (*light green*). θ is evaluated from AFM morphological images acquired using sharp pyramidal AFM probes with $R \approx 8$ nm (Figs. 2 *a* and S1 *a*). The estimation of the contact angle and condensate height derived from epifluorescence maps, expressed in nm/counts (Fig. 1 *a*), are both calibration routines performed with such a sharp pyramidal probe before assessing surface tension and viscosity using colloidal probes in an FRAP-ID experiment. With accurate calibrations, multiple experimental sessions can be performed without the need for recalibration, allowing for the exclusive use of colloidal probes. We use Eq. 1 to fit the retraction part of single indentation cycles performed with different colloidal probes (5, 6.62, and 10.2 μm diameters) during independent experiments, imposing γ and d as free fit parameters. Droplets with sizes ranging from few hundreds of nanometers to few micrometers were used.

FRAP-ID viscosity (η)

AFM-FS is employed with different AFM colloidal probe sizes (3.5, 5, and 10.2 μm in diameter) exerting a high force (>10–20 nN) on regions wetted by droplets larger than 10 μm, whose thickness ranged from 1 to 3 μm (measured by AFM). Fast probe retract leads to local dewetting, associated with the formation of a micrometric hole (dry spot) within the condensed phase. Rewetting is monitored by epifluorescence microscopy until complete fluorescence recovery (Fig. 1 *c*). The closure of dry spots within liquid films has already been investigated in the literature at the macroscale (millimetric regime) (51,52). A lubrication model describes the fluid flow in terms of variation of the liquid depth (h) over time (t) in function of γ, liquid density, and gravity acceleration (51,52,54). We specifically address the boundary condition at the edges of the dry spot, relating the speed of the contact line velocity $\delta a/\delta t$ to the static and dynamic contact angles (θ and $\delta h/\delta r$, respectively), using Tanner's law (51,55–57), as shown by

$$\frac{\delta a}{\delta t} = \frac{\gamma \omega}{2\eta}\left[\theta^3 - \left(\frac{\delta h}{\delta r}\right)^3\right], \tag{2}$$

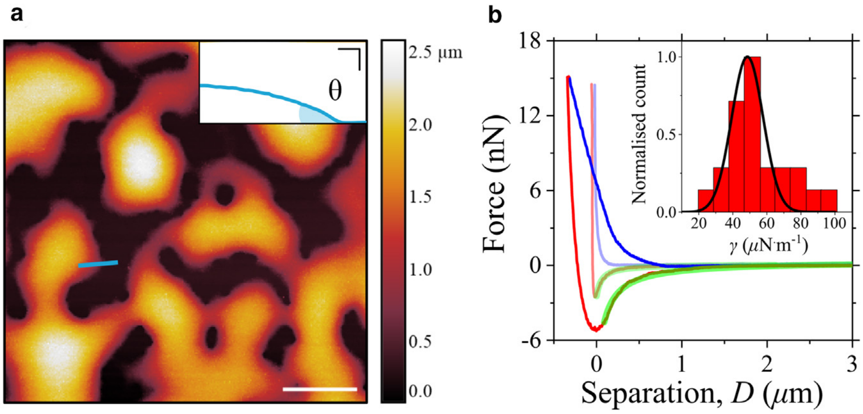

FIGURE 2 (*a*) AFM topography image of GFP-tagged ELF3 biomolecular condensates wetting a glass coverslip. Scale bar, 10 μm. The static contact angle (θ), shown in the inset (scale bars on both x and y axis = 1 μm), is determined along the profile (*light blue*) in the AFM image. (*b*) Two indentation cycles, performed with a colloidal probe (R = 2.5 μm) onto different droplets, are shown with both approach (*blue*) and retract (*red*). Indentations exhibit different hysteresis, reflecting the different size of the droplets. Retract curves are fitted (*green*) using Eq. 1, providing the droplet surface tension, whose distribution and the associated Gaussian fit (*black*) are shown in the inset.

where a is the radius of the dry spot, ω stands for the constant mobility of the contact line during hole collapse, and h represents the profile of the fluid depth, with $\delta h/\delta r$ its derivative at the dry spot edges, which can be considered as the dynamic contact angle (Fig. 1 *c*). In this study we do not numerically solve the equation described in the lubrication model. Instead, we experimentally characterize $\delta h/\delta r$ through epifluorescence frames acquired during rewetting (Fig. 1 *c*). To achieve this, fluorescence intensity is converted to fluid depth/thickness (h) upon calibration (for detailed protocol, see supporting material) and used to infer both $\delta h/\delta r$ through numerical differentiation and the dry spot radius a using a threshold set to 10% of the initial h. Additionally, we track the larger hole radius A, determined with a threshold set to 90% of the initial h, to monitor complete fluorescence recovery, which requires longer times than substrate rewetting. Therefore, we distinguish two regimes in our dataset. 1) Regime I is the focus of FRAP-ID. It is characterized by a nearly constant $\delta h/\delta r$, resulting in the decrease of $a(t)$ until hole collapse and complete rewetting ($a = 0$). Equation 2 holds in regime I, providing a reliable value of η, upon prior determination of θ and γ, in addition to the evaluation of the averaged $\delta h/\delta r$ across the regime. To achieve this, we normalize Eq. 2 by considering only the time of the experiment (defined by the critical time t_c), hence imposing $\omega = 1$. 2) Regime II is characterized by the recovery of the initial fluorescence achieved through the relaxation of the perturbed fluid morphology, leading to $A = 0$ (Fig. 1 *c*, *bottom*). Assuming the vertical fluid perturbation to be smaller than its characteristic in-plane length scale, the lubrication (thin film) model describing regime II relaxation is well described by (58,59):

$$\partial_t h + \frac{\gamma}{3\eta}\nabla \cdot \left[h^3 \nabla\left(\nabla^2 h\right)\right] = 0. \quad (3)$$

Given the circular spot within the fluid, Eq. 3 is invariant with the vertical axis and can be solved in an axisymmetric geometry, upon non-dimensionalization through $H = h/h_i$, $R = r/h_i$, and $T = \frac{\gamma t}{3\eta h_i}$, where h_i is the initial fluid depth, obtaining

$$\partial_T H + \frac{1}{R}\partial_R \left[RH^3\left(\partial_R^3 H + \frac{1}{R}\partial_R^2 H - \frac{1}{R^2}\partial_R H\right)\right] = 0. \quad (4)$$

Numerical simulations, conducted using a Runge-Kutta two-steps method, employ an experimental $h(r)$ profile from regime II as initial condition. Mass conservation is imposed as a boundary condition while the fixed parameter η/γ is set from FRAP-ID and AFM-FS experiments. Comparison with regime II profiles is then carried out over the experimental time t.

RESULTS

Liquid droplet formation of the ELF3 protein is induced by a decrease in pH and dilution to prevent collisions with the AFM probe (44) (see supporting material for the detailed protocol). The static contact angle is evaluated from the AFM topography of several condensates (Fig. 2 *a*), acquired using a sharp AFM tip, and resulting in $\theta = 31° \pm 10°$. The latter was estimated using multiple morphological profiles across several droplets (reported as *light-blue segments* in Figs. 2 *a* and S1 *a*), leading to a contact angle population reported in Fig. S1 *b*. It is noteworthy that the Young-Laplace equation could estimate the surface tension from Fig. 2 *a* if the differential pressure (or density) between the condensed and dilute phases were known.

Fig. 2 *b* shows two indentation cycles performed onto two different droplets. The retract part and the associated best fit using Eq. 1 are shown in red and green, respectively. The surface tension distribution for the 5-μm-diameter probe, made of borosilicate glass, is reported in the inset of Fig. 2 *b*, returning a value of 49 $\pm$ 9 μN $\cdot$ m^{-1}, corresponding to mean $\pm$ SD, and compatible with surface tension of other LLPS systems determined with coalescence experiments, OT, and other AFM experiments (2,15,17,41). The histograms related to tip diameters 6.62 and 10.2 μm, made of silicon dioxide, are reported in Fig. S2, providing a surface tension in agreement with the data shown in Fig. 2 *b*, and suggesting that droplets similarly wet silicon dioxide and glass. The concurrent coalescence of droplets and the gradual wetting of the substrate, both observed through epifluorescence, alongside complete FRAP within a few minutes (Fig. S3), suggest that the droplets were in a liquid state throughout each AFM experimental session. However, the droplet mechanical response changes after $\approx$30 min post LLPS, resulting in indentation cycles exhibiting force steps during retraction, possibly due to intermolecular rupture events, which we interpret as due to transition to a gel state (44) (Fig. S1 *c*). Over time, droplets fuse and spread across the glass coverslip, resulting in the formation of larger condensates (films). Indentations at a

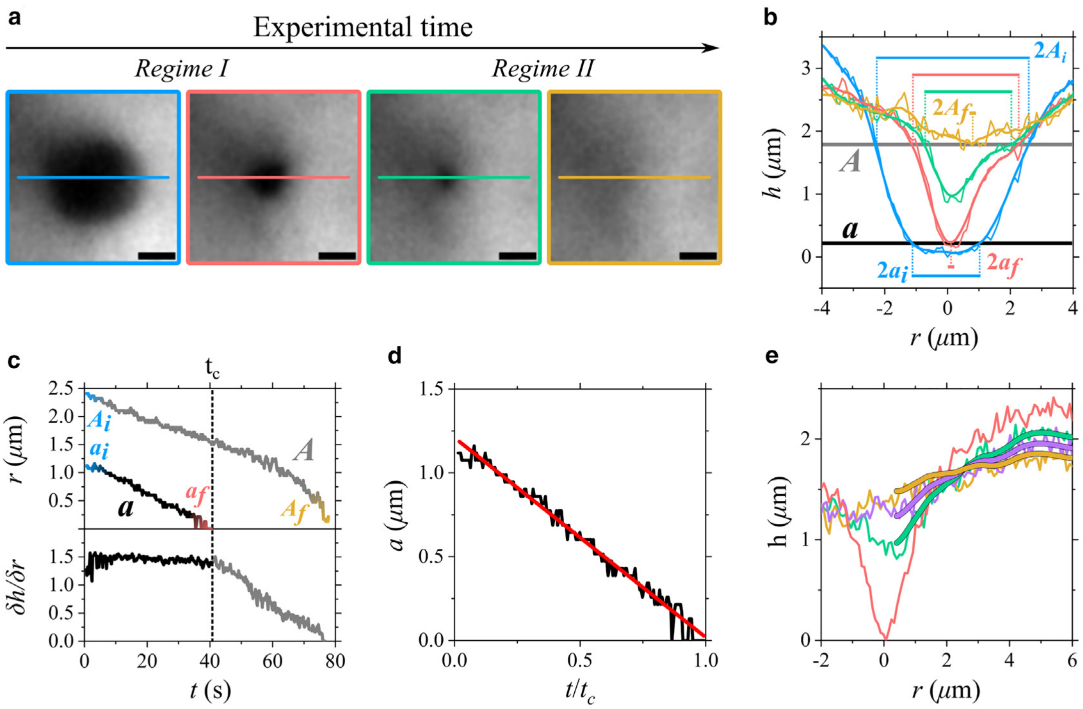

FIGURE 3 (*a*) Probe-induced dry spot exhibiting complete rewetting, from formation (*blue*) to closure (*yellow*) over time, observed through epifluorescence. Scale bars, 2 μm. In each panel, the segment used to extract the geometric parameters is shown. (*b*) Hole geometrical profiles, color-coded as the segments in (*a*): the intercept with threshold values are used to determine the dry spot radius a (*black horizontal line*), the corresponding larger hole radius A (*gray horizontal line*), and the edge $\delta h/\delta r$. The intersection of such thresholds with the profiles defines the initial and final hole radii. Subscripts i and f, for A and a, denote the initial and final values, respectively. (*c*) Evolution of A, a (*top*), and $\delta h/\delta r$ (*bottom*) during the time of the experiment. Complete rewetting occurs at $t = t_c$, when $a \approx 0$ (end of regime I, highlighted in boldface $\delta h/\delta r$). Regime II ($t > t_c$) is characterized by the recovery of the initial fluorescence through the relaxation of the fluid perturbation as tracked by A over time. (*d*) Evolution of hole radius a (*black*) as a function of the normalized time and associated best linear trend (*red*), providing an estimated viscosity. (*e*) Good agreement observed between numerical simulations (*thick lines*) and experimental profiles from regime II (*thin lines*) over a duration of 28 s.

high force induce dry spots followed by rewetting, whose timescale (tens of seconds) is reported in Fig. 3 and Video S1. The dry-spot profiles observed at different rewetting stages are plotted in Fig. 3 *b*. The corresponding dry spot radius (a), the larger radius (A), and $\delta h/\delta r$ from each frame are plotted over time in Fig. 3 *c*. Upon determination of $\delta h/\delta r$ during rewetting in regime I, the decrease of a with time leads to the evaluation of η over the time of the experiment t_c (Fig. 3 *d*). The value of viscosity obtained (24 $\pm$ 14 Pa $\cdot$ s, corresponding to mean $\pm$ SD) is close to values reported by passive microrheology studies for other LLPS condensates (17). Complete fluorescence recovery is achieved within 1–3 min in regime II, tracked through the larger radius A and returning the initial fluid depth h. In this scenario, the observed timescale for fluorescence recovery is inferior to the timescale measured in FRAP experiments (Fig. S3 and supporting text). This discrepancy is potentially due to the fact that FRAP-ID data account for the entire mass flow, including confined populations that do not contribute to conventional FRAP experiments. Additional reasons might include the presence of multimers or particles of heterogeneous sizes and situations where labeling is not uniform, all of which can significantly impact FRAP data and, potentially, fluorescence correlation spectroscopy as well.

Additionally, we observed several asymmetric closures due to the formation of dry spots with different $\delta h/\delta r$ along the contact line: an averaged $\delta h/\delta r$ was considered in these cases. If gravity and viscous forces are equilibrated within the fluid, the hole radius $a(t)$ is proportional to $(t_c - t)^{\delta}$, with $\delta \approx 1/10$, as reported by Tanner in his first works (60). However, important deviations have been documented in the literature, and $\delta \approx 0.76$ was observed by macroscopic studies (52,54). Our datasets indicate $0.7 < \delta < 0.95$ during rewetting. For $\delta = 0.95$ (Fig. 3 *e*), $\delta h/\delta r$ remains almost constant, and a nearly linear decrease of a with time is observed in regime I (Fig. 3 *c*). For lower δ values, we observed a gradual increase of $\delta h/\delta r$ with time, leading us to estimate the viscosity through linear approximations of $a(t)$ and $\delta h/\delta r$ over a timescale shorter than the full time of the experiment. Comparison between fluid relaxation in regime II and numerical simulations carried out using Eq. 4 demonstrates a good agreement. An illustrative example is presented in Fig. 3 *e* over a period of 28 s, assuming an inverse capillary velocity of 0.49 $s \cdot \mu m^{-1}$, as evaluated through FRAP-ID and AFM-FS. The initial $h(r)$

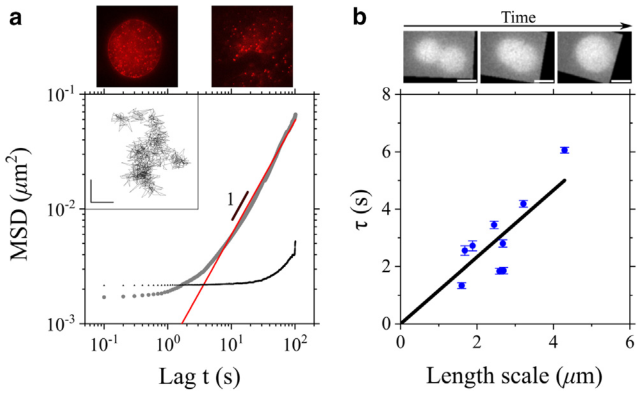

FIGURE 4 (*a*) Evaluation of droplet viscosity using passive microrheology. 50-nm (*left*) and 180-nm (*right*) beads embedded within protein condensates (*top*). The MSD vs. Lag *t* (*gray*) for 180-nm beads is fitted with a linear trend (*red*) to determine slope and intercept, the latter providing the diffusion coefficient. The instrumental background noise (*black*) is characterized by observing immobile particles in a separate experiment. The inset shows a particle trajectory. Scales bars on both x and y axis = 100 nm. (*b*) Coalescence between two droplets (*top*). Characteristic fusion time (τ) plotted against the droplet length scale (*blue circles*), and estimated linear trend (*black line*). Error bars derived from the exponential fit are shown.

(shown in *green*) is selected to ensure a vertical perturbation smaller than the in-plane perturbation length scale, a condition not met by the first profile of regime II (shown in *red*). Alternatively, one can iteratively adjust η/γ to achieve the best match between Eq. 4 numerical solutions and experiments. This approach allows for another evaluation of the η/γ parameter. Therefore, in summary, a single FRAP-ID measurement enables the assessment of η/γ (or directly η if γ is known from AFM-FS) through regime I, while regime II provides an independent re-evaluation of this parameter.

To further validate our data, we compare the viscosity estimated through FRAP-ID with data from both microrheological experiments (Videos S2 and S3) and coalescence experiments (Video S4). Fig. 4 *a* reports a typical mean square displacement (MSD) as a function of lag time in a log-log plot, allowing the determination of the type of motion ($\alpha = 1$ indicates Brownian motion), alongside the related fit that excludes the instrumental background noise (Fig. 4 *a* and supporting material). Considering curves with $0.9 < \alpha < 1.1$, the extrapolated diffusion coefficients lead to 34 Pa $\cdot$ s and 17 Pa $\cdot$ s for 50-nm- and 180-nm-diameter beads, respectively. Discrepancy may arise from variations in bead size and their interaction with the internal structures of the condensate, factors that can influence bead motion (36). Additionally, we observe a decrease of α with time, suggesting the rise of more confined trajectories. In agreement with the evolution of the force curves acquired by AFM, we ascribe this behavior to the liquid-to-gel state transition (droplet aging), partially accelerated by continuous exposure to the excitation laser used for epifluorescence imaging. Monitoring droplets coalescence across nine fusion events in separate experimental sessions provides the characteristic fusion time as a function of droplet length scale (Figs. 4 *b* and S5). The resulting inverse capillary velocity $\eta/\gamma = 1.1 \pm 0.2$ s $\cdot$ μm^{-1} is higher than the value obtained through AFM-FS and FRAP-ID, returning $\eta/\gamma = 0.49 \pm 0.30$ s $\cdot$ μm^{-1}. The discrepancy can be attributed to several factors: 1) during coalescence, droplets are observed in solution, whereas in FRAP-ID they interact with the substrate; 2) coalescence experiments were conducted under slightly different protein concentration and salt conditions compared to those used for FRAP-ID. Such a difference was introduced to increase the probability to detect coalescence events within the camera's field of view (see supporting material).

DISCUSSION

The sequential assessment of γ and η in a single experiment is a significant advantage, albeit subject to certain technical limitations. AFM-FS can estimate the surface tension of small droplets (<1 μm) that cannot be characterized with other methods, down to a few μN $\cdot$ m^{-1}. It is suitable for viscous biomolecular condensates of variable size and nature/composition, exhibiting sufficient attractive forces ($\geq$100 pN) to be measured by AFM. Indeed, optimization and increase of the AFM probe size might be necessary to enable the evaluation of a surface tension down to $\approx$1 μN $\cdot$ m^{-1}. FRAP-ID can be applied to dry spots with a size consistently larger than the diffraction limit, facilitating an accurate radius estimation. It is suited for condensates characterized by micrometric sizes, larger than the diameter of the tip used for dewetting, a condition which is usually reached after multiple coalescence events followed by wetting of the substrate. Dry spots collapsing within 4 s, observed through 21 frames at a rate of 5 frames per second, represent the fastest rewetting events we could properly analyze. Faster acquisition time can be beneficial only if fluorescence intensity signal-to-noise ratio is preserved. Therefore, for viscosities inferior to 1 Pa $\cdot$ s, the formation of larger dry spots is required, eventually using mechanical methods alternative to AFM. However, even in the absence of dry spot formation, low-viscosity condensates can be investigated using the lubrication analysis (regime II) introduced in this study. Regarding the accuracy of the FRAP-ID method, given the constant values for surface tension and contact angle used to estimate viscosity via Tanner's law (Eq. 2), we attribute the resulting uncertainty primarily to the fluorescence intensity noise affecting the pixels of the acquisition camera ($\approx$5%–10%). When propagated, this factor contributes significantly (15%–30%) to the statistical error reported for viscosity. Increasing the acquisition time for each fluorescence frame can effectively reduce the noise; however, this approach is feasible only for biomolecular condensates exhibiting slow dynamics.

Finally, it is worth noting the important deviation observed for δ in comparison with the expected value

reported for Tanner's law. In addition to cited cases (52,54), higher δ values have been reported for highly viscous fluids at early wetting stages (1/2 and 2/3). These observations are supported by experimental findings (61) and numerical simulations (62). Moreover, higher δ values have been reported for droplets spreading over rough surfaces and, importantly, for specific non-Newtonian fluids ($\delta = 1$) (63). In the latter case, the formation of surface tension gradients is hypothesized to be caused by the presence of slow-moving molecules that cannot rapidly migrate to the newly created interface. The biomolecular condensates studied in this work possess a heterogeneous molecular composition. The presence of molecules exhibiting confined diffusion, in addition to static molecular populations, has already been observed in our previous work (44). Therefore, this heterogeneity can potentially lead to non-Newtonian fluid behavior, resulting in an increased δ. In perspective, comparison of our data with different LLPS systems can potentially provide new perspectives on the dynamics of biomolecular condensates, highlighting fluid mechanics via FRAP-ID as a candidate to evaluate the Newtonian nature of the liquid condensate phase.

CONCLUSION

The methodology described in this paper offers the advantage of evaluating γ and η of biomolecular condensates in one single experiment, avoiding substrate functionalization and internalization of fluorescent beads, which is required for inverse capillary velocity measurements and passive microrheology, respectively. Moreover, this approach is particularly suitable for droplets with elevated surface tension in the $\mathrm{mN \cdot m^{-1}}$ range, where AFM excels. Therefore, it offers a valuable alternative to observing coalescence events, which are often hindered by the rapidity of the process. We have shown that the force exerted by a droplet on a micrometric spherical probe can be used to characterize the surface tension in the tens of $\mu\mathrm{N \cdot m^{-1}}$ range, and the same probe can be used thereafter to locally induce the formation of a dry spot, whose subsequent rewetting can be monitored to extract droplet viscosity in the tens of $\mathrm{Pa \cdot s}$ range. In this frame, rewetting is observed by tracking the fluorescence recovery due to the flow of GFP-tagged biomolecules within the condensed phase. A lubrication analysis of the subsequent fluid morphological relaxation can independently quantify the inverse capillary velocity, thereby cross-validating the values obtained from rewetting. This demonstrates the robustness of FRAP-ID. Furthermore, our findings are in good agreement with values obtained with passive microrheology, providing additional validation for this approach. Moreover, the viscosity assessed by FRAP-ID is a macroscopic property and hence independent of the presence of microdomains, which could influence the motion of fluorescent beads during microrheological experiments. The use of a simplified in vitro system to study and quantify LLPS parameters is an important first step in understanding the behavior of condensates and the physical characteristics of compartmentalized biological macromolecules. FRAP-ID is generally applicable to any in vitro LLPS-forming material, including synthetic polymers and complex mixtures of biological macromolecules. Indeed, our methodology can extend to broader applications, including nonbiological liquid-liquid interfaces. In these scenarios, surface tension and viscosity of micrometer-sized droplets vary depending on whether self-assembled materials decorate the interface. While quantifying these variations using conventional techniques at small micrometric scales may present challenges, FRAP-ID in combination with AFM-FS provides viable alternatives.

SUPPORTING MATERIAL

Supporting material can be found online at https://doi.org/10.1016/j.bpj.2024.07.043.

AUTHOR CONTRIBUTIONS

A.S., C.M.D., C.Z., P.-E.M., and L.C. designed the research. A.S. and S.H. carried out protein purification. A.S. performed all experiments and analyzed the data. L.C. carried out simulations. All authors wrote the article.

ACKNOWLEDGMENTS

The authors thank Luca Ciandrini, Yvonne Stahl and Manouk Abkarian for their support, including enriching scientific discussions and helpful feedback.

This project received support from the French National Research Agency (ANR) (ANR-19-CE20-0021 and ANR-21-CE11-0037). Additionally, we acknowledge the support from CNRS Momentum program (2017) and from the Plan Cancer Equipment 2016. The CBS is a member of France-BioImaging (FBI), a national infrastructure supported by the ANR (ANR-10-INBS-04-01), and of the GIS IBiSA (Infrastructures en Biologie Santé et Agronomie).

DECLARATION OF INTERESTS

The authors declare no competing interests.

SUPPORTING CITATIONS

References (64–75) appear in the supporting material.

REFERENCES

1. Banani, S. F., H. O. Lee, …, M. K. Rosen. 2017. Biomolecular condensates: organizers of cellular biochemistry. *Nat. Rev. Mol. Cell Biol.* 18:285–298. https://www.nature.com/articles/nrm.2017.7.

2. Brangwynne, C. P., C. R. Eckmann, …, A. A. Hyman. 2009. Germline P granules are liquid droplets that localize by controlled dissolution/condensation. *Science.* 324:1729–1732. https://doi.org/10.1126/science.1172046.

3. Liu, Q., J. Li, …, D. Zhou. 2021. Glycogen accumulation and phase separation drives liver tumor initiation. *Cell.* 184:5559–5576.e19. https://doi.org/10.1016/j.cell.2021.10.001.

4. Gao, Y., X. Li, …, Y. Lin. 2022. A brief guideline for studies of phase-separated biomolecular condensates. *Nat. Chem. Biol.* 18:1307–1318. https://doi.org/10.1038/s41589-022-01204-2.

5. Brangwynne, C. P., T. J. Mitchison, and A. A. Hyman. 2011. Active liquid-like behavior of nucleoli determines their size and shape in Xenopus laevis oocytes. *Proc. Natl. Acad. Sci. USA.* 108:4334–4339. https://doi.org/10.1073/pnas.1017150108.

6. Li, P., S. Banjade, …, M. K. Rosen. 2012. Phase transitions in the assembly of multivalent signalling proteins. *Nature.* 483:336–340. https://www.nature.com/articles/nature10879.

7. Banjade, S., and M. K. Rosen. 2014. Phase transitions of multivalent proteins can promote clustering of membrane receptors. *Elife.* 3:e04123. https://doi.org/10.7554/eLife.04123.

8. Kato, M., T. W. Han, …, S. L. McKnight. 2012. Cell-free formation of RNA granules: Low complexity sequence domains form dynamic fibers within hydrogels. *Cell.* 149:753–767. https://www.cell.com/cell/abstract/S0092-8674(12)00514-4.

9. Shin, Y., and C. P. Brangwynne. 2017. Liquid phase condensation in cell physiology and disease. *Science.* 357:eaaf4382. https://doi.org/10.1126/science.aaf4382.

10. Bouchard, J. J., J. H. Otero, …, T. Mittag. 2018. Cancer Mutations of the Tumor Suppressor SPOP Disrupt the Formation of Active, Phase-Separated Compartments. *Mol. Cell.* 72:19–36.e8. https://doi.org/10.1016/j.molcel.2018.08.027.

11. Alberti, S., and D. Dormann. 2019. Liquid–Liquid Phase Separation in Disease. *Annu. Rev. Genet.* 53:171–194.

12. Elbaum-Garfinkle, S. 2019. Matter over mind: Liquid phase separation and neurodegeneration. *J. Biol. Chem.* 294:7160–7168. https://doi.org/10.1074/jbc.REV118.001188.

13. Spruijt, E. 2023. Open questions on liquid–liquid phase separation. *Commun. Chem.* 6:23. https://doi.org/10.1038/s42004-023-00823-7.

14. Darling, A. L., and J. Shorter. 2021. Combating deleterious phase transitions in neurodegenerative disease. *Biochim. Biophys. Acta Mol. Cell Res.* 1868:118984. https://www.sciencedirect.com/science/article/pii/S0167488921000380.

15. Li, X., J. van der Gucht, …, R. de Vries. 2023. Active microrheology of protein condensates using colloidal probe-AFM. *J. Colloid Interface Sci.* 632:357–366. https://doi.org/10.1016/j.jcis.2022.11.071.

16. Viola, G., F. Floriani, …, M. Assfalg. 2023. Ultrasmall Gold Nanoparticles as Clients of Biomolecular Condensates. *Chem. Eur J.* 29:e202301274. https://doi.org/10.1002/chem.202301274.

17. Fisher, R. S., and S. Elbaum-Garfinkle. 2020. Tunable multiphase dynamics of arginine and lysine liquid condensates. *Nat. Commun.* 11:4628. https://doi.org/10.1038/s41467-020-18224-y.

18. Elbaum-Garfinkle, S., Y. Kim, …, C. P. Brangwynne. 2015. The disordered P granule protein LAF-1 drives phase separation into droplets with tunable viscosity and dynamics. *Proc. Natl. Acad. Sci. USA.* 112:7189–7194. https://doi.org/10.1073/pnas.1504822112.

19. Sawyer, I. A., D. Sturgill, and M. Dundr. 2019. Membraneless nuclear organelles and the search for phases within phases. *WIREs RNA.* 10:e1514. https://doi.org/10.1002/wrna.1514.

20. Holehouse, A. S., and R. V. Pappu. 2018. Functional Implications of Intracellular Phase Transitions. *Biochemistry.* 57:2415–2423. https://doi.org/10.1021/acs.biochem.7b01136.

21. Mathieu, C., R. V. Pappu, and J. P. Taylor. 2020. Beyond aggregation: Pathological phase transitions in neurodegenerative disease. *Science.* 370:56–60. https://doi.org/10.1126/science.abb8032.

22. Linsenmeier, M., M. Hondele, …, P. Arosio. 2022. Dynamic arrest and aging of biomolecular condensates are modulated by low-complexity domains, RNA and biochemical activity. *Nat. Commun.* 13:3030. https://doi.org/10.1038/s41467-022-30521-2.

23. Feric, M., N. Vaidya, …, C. P. Brangwynne. 2016. Coexisting Liquid Phases Underlie Nucleolar Subcompartments. *Cell.* 165:1686–1697. https://doi.org/10.1016/j.cell.2016.04.047.

24. Wang, J., J. M. Choi, …, A. A. Hyman. 2018. A Molecular Grammar Governing the Driving Forces for Phase Separation of Prion-like RNA Binding Proteins. *Cell.* 174:688–699.e16. https://doi.org/10.1016/j.cell.2018.06.006.

25. Taylor, N. O., M. T. Wei, …, C. P. Brangwynne. 2019. Quantifying Dynamics in Phase-Separated Condensates Using Fluorescence Recovery after Photobleaching. *Biophys. J.* 117:1285–1300. https://doi.org/10.1016/j.bpj.2019.08.030.

26. Erdel, F., A. Rademacher, …, K. Rippe. 2020. Mouse Heterochromatin Adopts Digital Compaction States without Showing Hallmarks of HP1-Driven Liquid-Liquid Phase Separation. *Mol. Cell.* 78:236–249.e7. https://doi.org/10.1016/j.molcel.2020.02.005.

27. Muzzopappa, F., J. Hummert, …, F. Erdel. 2022. Detecting and quantifying liquid–liquid phase separation in living cells by model-free calibrated half-bleaching. *Nat. Commun.* 13:7787. https://doi.org/10.1038/s41467-022-35430-y.

28. Alshareedah, I., T. Kaur, …, P. R. Banerjee. 2019. Interplay Between Short-range Attraction and Long-range Repulsion Controls Reentrant Liquid Condensation of Ribonucleoprotein-RNA Complexes. *J. Am. Chem. Soc.* 141:14593–14602. https://doi.org/10.1021/jacs.9b03689.

29. Kaur, T., I. Alshareedah, …, P. R. Banerjee. 2019. Molecular Crowding Tunes Material States of Ribonucleoprotein Condensates. *Biomolecules.* 9:71. https://doi.org/10.3390/biom9020071.

30. Alshareedah, I., T. Kaur, and P. R. Banerjee. 2021. Methods for Characterizing the Material Properties of Biomolecular Condensates. *Methods Enzymol.* 646:143–183. https://doi.org/10.1016/bs.mie.2020.06.009.

31. Patel, A., H. O. Lee, …, S. Alberti. 2015. A Liquid-to-Solid Phase Transition of the ALS Protein FUS Accelerated by Disease Mutation. *Cell.* 162:1066–1077. https://doi.org/10.1016/j.cell.2015.07.047.

32. Alshareedah, I., G. M. Thurston, and P. R. Banerjee. 2021. Quantifying viscosity and surface tension of multicomponent protein-nucleic acid condensates. *Biophys. J.* 120:1161–1169. https://doi.org/10.1016/j.bpj.2021.01.005.

33. Alberti, S., A. Gladfelter, and T. Mittag. 2019. Considerations and Challenges in Studying Liquid-Liquid Phase Separation and Biomolecular Condensates. *Cell.* 176:419–434. https://doi.org/10.1016/j.cell.2018.12.035.

34. Jawerth, L., E. Fischer-Friedrich, …, F. Jülicher. 2020. Protein condensates as aging Maxwell fluids. *Science.* 370:1317–1323. https://doi.org/10.1126/science.aaw4951.

35. Coupe, S., and N. Fakhri. 2024. ATP-induced cross-linking of a biomolecular condensate. *Biophys. J.* 123:1356–1366. https://doi.org/10.1016/j.bpj.2023.07.013.

36. Feric, M., and C. P. Brangwynne. 2013. A nuclear F-actin scaffold stabilizes RNP droplets against gravity in large cells. *Nat. Cell Biol.* 14:1253–1259. https://doi.org/10.1038/ncb2830.

37. Ghosh, A., D. Kota, and H.-X. Zhou. 2021. Shear relaxation governs fusion dynamics of biomolecular condensates. *Nat. Commun.* 12:5995. https://doi.org/10.1038/s41467-021-26274-z.

38. Sprakel, J., N. A. M. Besseling, …, M. A. Cohen Stuart. 2007. Equilibrium capillary forces with atomic force microscopy. *Phys. Rev. Lett.* 99:104504–104505. https://doi.org/10.1103/PhysRevLett.99.104504.

39. Spruijt, E., J. Sprakel, …, J. Van Der Gucht. 2010. Interfacial tension between a complex coacervate phase and its coexisting aqueous phase. *Soft Matter.* 6:172–178. https://doi.org/10.1039/B911541B.

40. Lee, H. D., and A. V. Tivanski. 2021. Atomic force microscopy: an emerging tool in measuring the phase state and surface tension of individual aerosol particles. *Annu. Rev. Phys. Chem.* 72:235–252. https://doi.org/10.1146/annurev-physchem-090419-110133.

41. Jawerth, L. M., M. Ijavi, …, E. Fischer-Friedrich. 2018. Salt-Dependent Rheology and Surface Tension of Protein Condensates Using Optical Traps. *Phys. Rev. Lett.* 121:258101. https://doi.org/10.1103/PhysRevLett.121.258101.

42. Ceballos, A. V., C. J. McDonald, and S. Elbaum-Garfinkle. 2018. Methods and Strategies to Quantify Phase Separation of Disordered Proteins. *Methods Enzymol.* 611:31–50. https://doi.org/10.1016/bs.mie.2018.09.037.

43. Fernandes, T. F. D., O. Saavedra-Villanueva, …, L. Costa. 2020. Synchronous, Crosstalk-free Correlative AFM and Confocal Microscopies/Spectroscopies. *Sci. Rep.* 10:7098. https://doi.org/10.1038/s41598-020-62529-3.

44. Hutin, S., J. R. Kumita, …, C. Zubieta. 2023. Phase separation and molecular ordering of the prion-like domain of the Arabidopsis thermosensory protein EARLY FLOWERING 3. *Proc. Natl. Acad. Sci. USA.* 120:e2304714120. https://doi.org/10.1073/pnas.2304714120.

45. Dahmane, S., C. Doucet, …, P. E. Milhiet. 2019. Nanoscale organization of tetraspanins during HIV-1 budding by correlative dSTORM/AFM. *Nanoscale.* 11:6036–6044. https://pubs.rsc.org/en/content/articlehtml/2019/nr/c8nr07269h.

46. Vial, A., L. Costa, …, C. M. Doucet. 2023. Structure and mechanics of the human nuclear pore complex basket using correlative AFM-fluorescence superresolution microscopy. *Nanoscale.* 15:5756–5770. https://doi.org/10.1039/D2NR06034E.

47. Elena-Real, C. A., A. Sagar, …, P. Bernadó. 2023. The structure of pathogenic huntingtin exon 1 defines the bases of its aggregation propensity. *Nat. Struct. Mol. Biol.* 30:309–320. https://www.nature.com/articles/s41594-023-00920-0.

48. Schatz, M., L. Marty, …, B. Beaumelle. 2023. A Tripartite Complex HIV-1 Tat-Cyclophilin A-Capsid Protein Enables Tat Encapsidation That Is Required for HIV-1 Infectivity. *J. Virol.* 97. e0027823–23. https://doi.org/10.1128/jvi.00278-23.

49. Odermatt, P. D., A. Shivanandan, …, G. E. Fantner. 2015. High-Resolution Correlative Microscopy: Bridging the Gap between Single Molecule Localization Microscopy and Atomic Force Microscopy. *Nano Lett.* 15:4896–4904. https://doi.org/10.1021/acs.nanolett.5b00572.

50. Israelachvili, J. N. 2011. Intermolecular and Surface Forces, third edition. Elsevier Inc.

51. López, P. G., M. J. Miksis, and S. G. Bankoff. 2001. Stability and evolution of a dry spot. *Phys. Fluids.* 13:1601–1614. https://doi.org/10.1063/1.1369607.

52. Bankoff, S. G., M. F. G. Johnson, …, P. G. Lopez. 2003. Dynamics of a dry spot. *J. Fluid Mech.* 486:239–259. https://www.cambridge.org/core/journals/journal-of-fluid-mechanics/article/dynamics-of-a-dry-spot/286ED7EFB4B9161DF378D5EC80718647.

53. Jung, J. H., A. D. Barbosa, …, P. A. Wigge. 2020. A prion-like domain in ELF3 functions as a thermosensor in Arabidopsis. *Nature.* 585:256–260. https://doi.org/10.1038/s41586-020-2644-7.

54. Diez, J. A., R. Gratton, and J. Gratton. 1992. Self-similar solution of the second kind for a convergent viscous gravity current. *Phys. Fluid.* 4:1148–1155. https://doi.org/10.1063/1.858233.

55. Ehrhard, P., and S. H. Davis. 1991. Non-isothermal spreading of liquid drops on horizontal plates. *J. Fluid Mech.* 229:365–388. https://doi.org/10.1017/S0022112091003063.

56. Sellier, M., J. W. Grayson, …, A. K. Bertram. 2015. Estimating the viscosity of a highly viscous liquid droplet through the relaxation time of a dry spot. *J. Rheol. (N. Y., NY, U. S.).* 59:733–750. https://doi.org/10.1122/1.4917240.

57. McKinley, I. S., S. K. Wilson, and B. R. Duffy. 1999. Spin coating and air-jet blowing of thin viscous drops. *Phys. Fluids.* 11:30–47. https://doi.org/10.1063/1.869922.

58. Salez, T., J. D. McGraw, …, E. Raphaël. 2012. Numerical solutions of thin-film equations for polymer flows. *Eur. Phys. J. E Soft Matter.* 35:114. https://doi.org/10.1140/epje/i2012-12114-x.

59. Lakshman, S., W. Tewes, …, D. Lohse. 2021. Deformation and relaxation of viscous thin films under bouncing drops. *J. Fluid Mech.* 920:A3. https://doi.org/10.1017/jfm.2021.378.

60. Tanner, L. H. 1979. The spreading of silicone oil drops on horizontal surfaces. *J. Phys. D Appl. Phys.* 12:1473–1484. https://doi.org/10.1088/0022-3727/12/9/009.

61. Eddi, A., K. G. Winkels, and J. H. Snoeijer. 2013. Short time dynamics of viscous drop spreading. *Phys. Fluid.* 25:013102. https://doi.org/10.1063/1.4788693.

62. Legendre, D., and M. Maglio. 2013. Numerical simulation of spreading drops. *Colloids Surf. A Physicochem. Eng. Asp.* 432:29–37. https://doi.org/10.1016/j.colsurfa.2013.04.046.

63. Rafaï, S., and D. Bonn. 2005. Spreading of non-Newtonian fluids and surfactant solutions on solid surfaces. *Phys. Stat. Mech. Appl.* 358:58–67. https://doi.org/10.1016/j.physa.2005.06.017.

64. Vial, A., C. Taveneau, …, P. E. Milhiet. 2021. Correlative AFM and fluorescence imaging demonstrate nanoscale membrane remodeling and ring-like and tubular structure formation by septins. *Nanoscale.* 13:12484–12493. https://doi.org/10.1039/D1NR01978C.

65. Sader, J. E., R. Borgani, …, T. Zheng. 2016. A virtual instrument to standardise the calibration of atomic force microscope cantilevers. *Rev. Sci. Instrum.* 87:093711. https://doi.org/10.1063/1.4962866.

66. Proksch, R., T. E. Schäffer, …, M. B. Viani. 2004. Finite optical spot size and position corrections in thermal spring constant calibration. *Nanotechnology.* 15:1344–1350. https://doi.org/10.1088/0957-4484/15/9/039.

67. Nečas, D., and P. Klapetek. 2012. Gwyddion: An open-source software for SPM data analysis. *Cent. Eur. J. Phys.* 10:181–188. https://doi.org/10.2478/s11534-011-0096-2.

68. Dosset, P., P. Rassam, …, P. E. Milhiet. 2016. Automatic detection of diffusion modes within biological membranes using back-propagation neural network. *BMC Bioinf.* 17:197. https://doi.org/10.1186/s12859-016-1064-z.

69. Qian, H., M. P. Sheetz, and E. L. Elson. 1991. Single particle tracking. Analysis of diffusion and flow in two-dimensional systems. *Biophys. J.* 60:910–921. https://doi.org/10.1016/S0006-349591.82125-7.

70. Kusumi, A., Y. Sako, and M. Yamamoto. 1993. Confined lateral diffusion of membrane receptors as studied by single particle tracking (nanovid microscopy). Effects of calcium-induced differentiation in cultured epithelial cells. *Biophys. J.* 65:2021–2040. https://doi.org/10.1016/S0006-3495(93)81253-0.

71. Garting, T., and A. Stradner. 2019. Synthesis and application of PEGylated tracer particles for measuring protein solution viscosities using Dynamic Light Scattering-based microrheology. *Colloids Surf. B Biointerfaces.* 181:516–523. https://doi.org/10.1016/j.colsurfb.2019.05.059.

72. Daniels, B. R., B. C. Masi, and D. Wirtz. 2006. Probing single-cell micromechanics in vivo: The microrheology of C. elegans developing embryos. *Biophys. J.* 90:4712–4719. https://doi.org/10.1529/biophysj.105.080606.

73. Schneider, C. A., W. S. Rasband, and K. W. Eliceiri. 2012. NIH Image to ImageJ: 25 years of image analysis. *Nat. Methods.* 9:671–675. https://www.nature.com/articles/nmeth.2089.

74. Abràmoff, M. D., P. J. Magalhães, and S. J. Ram. 2004. Image processing with imageJ. *Biophot. Int.* 11:36–41.

75. Thévenaz, P., U. E. Ruttimann, and M. Unser. 1998. A pyramid approach to subpixel registration based on intensity. *IEEE Trans. Image Process.* 7:27–41. https://doi.org/10.1109/83.650848.

Biophysical Journal, Volume *123*

Supplemental information

Quantifying surface tension and viscosity in biomolecular condensates by FRAP-ID

Andreas Santamaria, Stephanie Hutin, Christine M. Doucet, Chloe Zubieta, Pierre-Emmanuel Milhiet, and Luca Costa

Supplemental Material

MATERIALS

Bis-Tris propane (1,3-bis(tris(hydroxymethyl)methylamino)propane, purity ≥ 99%) was purchased from Merck; TCEP (Tris(2-carboxyethyl)phosphine hydrochloride, purity ≥ 98%) was purchased from Roth; sodium chloride (NaCl, purity ≥ 99%) was purchased from Euromedex; imidazole (purity ≥ 99%) and potassium chloride (KOH, purity ≥ 85%) were purchased from Merk; tris base (Tris[hydroxymethyl]aminomethane, purity ≥ 98.8%) was purchased from Merk Biorad; Ni Sepharose 6 Fast Flow Cytiva was purchased from Merk.

PROTEIN EXPRESSION AND PURIFICATION

ELF3 prion-like domain (Q7, residues 388-625, AT2G25930, *Arabidopsis thaliana* ecotype Columbia), tagged with Green Fluorescent Protein (GFP), was expressed in *Escherichia coli* BL21-CodonPlus-RIL cells (Agilent), as previously described (1, 2). Cells were resuspended in lysis buffer (100 mM Bis-Tris propane pH 9.4, 300 mM NaCl, 20 mM imidazole, 1 mM TCEP, plus 1x protease inhibitors (Roche)), lysed via sonication, spun at 45.000 x g to remove cell debris and the supernatant applied to a Ni-NTA column (1 mL of Ni Sepharose pre-equilibrated with resuspension buffer). The column was then washed with 50 column volumes (CV) of lysis buffer followed by a high salt wash (50 CV) with 100 mM Bis-Tris propane pH 9.4, 1 M NaCl, 20 mM imidazole, 1 mM TCEP. Finally, the proteins were eluted in 100 mM Bis-Tris propane pH 9.4, 300 mM NaCl, 300 mM imidazole, and 1 mM TCEP. Protein purity was determined by SDS-PAGE. The final protein concentration in the fraction used for all the experiments was 12.4 mg $\cdot$ mL^{-1}, calculated using A280 absorption with a NanoDrop instrument (NanoDrop one, ThermoFisher).

PROTEIN DROPLETS FORMATION

LLPS was triggered by a decrease in the pH of the solution. 4 μL of the protein solution in 100 mM Bis-Tris propane pH 9.4, 300 mM NaCl, 300 mM imidazole, and 1 mM TCEP were diluted with 4 μL of a buffering solution made up of 100 mM Tris buffer pH 7.5. This 1:1 mixing lead to a final pH of ≈ 8.2. The solution was deposited onto a glass coverslip (25 mm diameter, 0.165 mm thick, purchased from Marienfeld), previously cleaned with KOH 1M for 15 minutes in a sonication bath, and then extensively rinsed and sonicated for 15 minutes with MilliQ water. In such a crowded solution, collisions between large droplets and the AFM cantilever occur. Such collisions must be limited to ensure proper AFM data acquisition (1). Therefore, once the appearance of droplets in the solution deposited on the coverslip was assessed through fluorescence microscopy, 40 μL of a mixture of 33 mM Bis-Tris propane and 33 mM Tris buffer (pH=8.5) were added to dilute the solution to a final protein concentration of 1 mg $\cdot$ mL^{-1}, 42 mM NaCl, 36 mM Tris, 36 mM BTP and 0.08 mM TCEP, which drastically reduces droplets-AFM cantilever collisions. All our data acquisitions were conducted under these conditions, except for the coalescence experiments. In this specific case, we omitted the final dilution with 40 μL of the mixture containing 33 mM Bis-Tris propane and 33 mM Tris buffer (pH=8.5). This exclusion increased the probability of observing coalescence events within the camera's field of view.

CORRELATIVE ATOMIC FORCE MICROSCOPY - WIDE FIELD FLUORESCENCE MICROSCOPY

AFM coupled with a wide-field fluorescence microscope was developed in-house, allowing simultaneously AFM and fluorescence imaging (1, 3–8). It is based on a Nanowizard 4 (JPK Instruments, Bruker) mounted on a Zeiss inverted optical microscope, equipped with a LX 488-50 OBIS (488 nm, Coherent), a Sapphire 561-100 CW (561 nm, Coherent) and a F-04306-107 (642 nm, MPB) as laser sources and an oil immersion objective with a 1.46 numerical aperture (Plan-Apochromat 100X, Zeiss). Fluorescence was collected with an EmCCD iXon Ultra897 (Andor) camera. An ET800sp short-pass filter (Chroma) was used in the emission optical path to filter out the light source of the AFM optical beam deflection system. Epifluorescence images were acquired using the emission filter ET525/50 nm (Chroma) and an acousto-optic tunable filter (AOTFnc-400.650-TN, AA opto-electronics) to modulate the laser intensity and set the exposure time for each frame, typically between 100 and 400 ms. The setup includes a telescope to reach a final imaging magnification corresponding to a camera pixel size of 86 nm, resulting in fluorescence images 44.032 μm x 44.032 μm large. The 488 nm laser excitation power was measured before the objective with a PM100 energy meter (purchased from Thorlabs) and was optimized in all the experiments in the range of 0.3-1 μW.

- AFM imaging and contact angle evaluation

To determine the condensate contact angle after deposition on the glass coverslip, AFM images were acquired in Quantitative Imaging (QI) mode with a scan size of 50 μm × 50 μm and with 512 lines x 512 pixels. Images were acquired with a maximal force of 3 nN over oscillation cycles (Z length) of 2 μm. We used AC40TS AFM cantilevers, purchased from Olympus. The inverse optical lever sensitivity and the cantilever spring constant were calibrated using a combination of Sader (9) and thermal methods (10) in liquid environment (called *contact-free* method in the JPK AFM). Image treatment was performed using Gwyddion (11). Several morphological profiles were taken into account (see Fig. 2(a) and Fig. S1(a)) and a linear fit was performed to estimate the profile slope in correspondence to the edge of each wetting region, considered as the contact angle. The outcomes were averaged using a normal distribution, resulting in θ=31°±10° (Fig. S1(b)).

- AFM-Force Spectroscopy: surface tension

AFM cantilevers with colloidal probes with diameters equal to 5 μm (CP-CONT-BSG-A), 6.62 μm (CP-qp-CONT-SiO-C) and 10.2 μm (CP-PNPL-SiO-D), were purchased from NanoAndMore GmbH. The optical lever sensitivity was calibrated by performing indentation cycles on a rigid glass coverslip, while the spring constant was evaluated using the thermal method (10). AFM-Force Spectroscopy (AFM-FS) was performed through indentation cycles with a maximal force setpoint of 15 nN over probe-sample distances ranging from 4 to 8 μm (Z length), depending on the size of the droplet investigated. Only force curves in which both the approach and retract parts were flat at high tip-sample distance (D) were fitted. Among these, only the ones that returned a value of 0.05 μm ≤ d ≤ 1 μm were taken into account for the final evaluation of δ, since Eq. 1 requires d to be much smaller than the probe radius. Moreover, spurious values of δ and also negligible secondary populations, probably due to condensate aging, were excluded (see Fig. S2). In particular, 85%, 81% and 92% of the total amount of the data were used for δ determination, for 5, 6.62 and 10.2 μm tip diameter, leading to 49 ± 9, 63 ± 23 and 37 ± 24 $\mu\mathrm{N} \cdot \mathrm{m}^{-1}$, respectively. Surface tension data were fitted with a Gaussian distribution, and the associated results are reported as x_c±w, where x_c is the mean value and w is the standard deviation. This study involved the analysis of approximately 200 indentation curves acquired over six independent experimental sessions.

- Fluid depth/Fluorescence intensity calibration

AFM morphological images can be used for the fluid depth/fluorescence intensity calibration. Fluorescence and AFM topographical images are finely aligned and superimposed. For each pixel, the fluid height (depth) value was divided by the corresponding fluorescence intensity value, returning the calibration parameter of 0.25 nm·count^{-1} which is constant over the central region of the fluorescence image (30 μm x 30 μm). This value was employed to determine the droplet thickness h used in the FRAP-ID analysis.

- Viscosity: Fluorescence Recovery After Probe-Induced Dewetting (FRAP-ID)

In all FRAP-ID experiments, the laser power was set to 0.3 μW (488 nm). Dry spots were formed using colloidal AFM probes with diameters equal to 3.5 μm (CP-PNPL-SiO-B), 5 μm (CP-CONT-BSG-A) and 10.2 μm (CP-PNPL-SiO-D), purchased from NanoAndMore GmbH. Fluorescence images of probe-induced dry spots, saved in a stack of individual frames, are cropped so that the first frame corresponds to the initial dewetting, ensuring that the AFM probe had been placed far from the indentation region to avoid spurious fluorescence and probe reflections. Multiple profiles across the holes are extracted using ImageJ, exporting fluorescence intensity at each pixel for all time-dependent frames. Calibration for fluid depth/fluorescence intensity (0.25 nm·count^{-1}) and length/pixel ratio (86 nm·pixel^{-1}) are applied, obtaining fluid depth vs length profiles (Fig. 3(b)). The latter are low-passed filtered and the hole radii a and A are then evaluated using height thresholds, corresponding to 10% and 90% of the hole depth, respectively (depicted in grey and black in Fig. 3(b)). The experimental critical time (t_c), defined as the time necessary for the hole to collapse, corresponding to complete rewetting ($a \approx 0$), is here estimated from the fluorescence images, leading to ω = 1. Then, a is plotted over the experimental time (Fig. 3(c) top). Subsequently, $\delta h/\delta r$ is determined along the segment upon numerical differentiation at the edge of the hole, and is plotted vs time (Fig. 3(c) bottom). The latter is averaged over the rewetting time (t_c). Imposing θ and δ as fixed fit parameters, when $a(t)$ is linearly fitted using Eq. 2 (Fig. 3(d)) to assess the viscosity. In our experiments, we recorded an image each 200 or 400 ms. We analyzed fluorescence recovery data from 27 independent rewetting datasets, collected during four separate experimental sessions.

FLUORESCENCE RECOVERY AFTER PHOTOBLEACHING (FRAP)

Measurements were carried out with a correlative AFM-confocal microscope described in refs (1, 8) equipped with a supercontinuum laser (20 MHz, Rock-PP, Leukos) as laser source and an oil immersion objective with a 1.4 NA (Plan-Apochromat 100X, Zeiss). Fluorescence was collected after a 100 μm diameter pin-hole (P100D, Thorlabs) by an avalanche photodetector (SPCM-AQR-15, PerkinElmer) connected to an SPC-150 (Becker & Hickl) TCSPC card. The excitation laser was filtered with a ZET488/10 filter (Chroma) and the associated power was measured at the sample level with a S170C microscope slide power sensor and a PM100 energy meter (both purchased from Thorlabs). The power was set to 0.1 μW to image droplets, and to 150 μW to induce photobleaching (exposure time = 1 s). Confocal images and FRAP were acquired using a 525/39 nm (Thorlabs) emission filter. FRAP datasets exhibit complete fluorescence recovery within 5-10 minutes (Fig. S3), slower than the timescale observed in FRAP-ID rewetting experiments (30 s - 1 minute), although comparable with regime II timescale (fluid relaxation). FRAP data could not be properly fitted considering a single population, suggesting the presence of diffusing entities of different sizes and showing the complexity of the LLPS system investigated in this study.

PASSIVE MICRORHEOLOGY

Fluorescent spheres with diameters of 50 nm (40 nm nominal, 540/560 nm, purchased from Invitrogen F8792) and 180 nm (170 nm nominal, 633/660 nm, purchased from Life technologies P7220) were used to assess condensate viscosity through passive microrheology, in separate experiments. A solution of beads in water was mixed to the protein solution before pH drop and LLPS triggering. The same fluorescence microscope previously described was used to visualize 30 to 90 embedded beads moving within each condensate. Data were acquired at 10 frames $\cdot$ s^{-1} with laser power equal to 240 μW and 3 μW, for 561 nm and 642 nm laser sources, respectively. Data were treated with PaTrack software (12), which calculates the mean square displacement (MSD) of the beads as a function of lag time (Fig. S4).

$$MSD(n\delta t) = \frac{1}{N-n-1} \sum_{j=1}^{N-n-1} \{[x(j\delta t + n\delta t) - x(j\delta t)]^2 - [y(j\delta t + n\delta t) - y(j\delta t)]^2\} \quad \text{(S1)}$$

where δt is the time interval between two successive frames (here 100 ms), N is the total number of frames (here $\approx$ 1000, corresponding to 100 s), n is the number of time intervals, $x(t)$ and $y(t)$ are the bead coordinates at time t (13, 14). The MSD vs t profiles were fitted using an exponential model MSD= $4Dt^{\alpha}$, where D is the diffusion coefficient, and α indicates the type of motion ($\alpha < 1$, confined or subdiffusive; α=1 Brownian; $\alpha > 1$ superdiffusive). The initial $\approx$ 20% of the dataset is situated within the instrumental background noise and, consequently, was not taken into account. The background noise was determined by evaluating the MSD of immobile beads deposited onto a glass coverslip (Fig. 4(a)).

Data were displayed in a log-log plot, and only the curves with $0.9 \leq \alpha \leq 1.1$ were considered to determine condensate viscosity. The diffusion coefficient was determined from the intercept, leading to the viscosity of 43 Pa·s and 17 Pa·s for 50 and 180 nm beads, respectively. We imaged each condensate for $\approx$ 400 s. Data were then divided into ensembles of 100 s and treated separately, We observed a slow decrease of α with time, suggesting the appearance of more confined trajectories, which we ascribe to the aging process of the droplets. Additionally, to prevent interaction of the beads with ELF3 proteins in the condensed phase, we prepared PEGylated beads, as described in refs (15, 16). However, to the best of our observations, PEGylated beads did not embed within ELF3 droplets. This suggests that a different beads functionalization strategy is required, contributing to the complexity of data acquisition and interpretation in passive microrheology (17, 18), as mentioned in the main manuscript. A total of 642 trajectories were observed across four distinct experimental sessions.

COALESCENCE EXPERIMENTS

Fluorescence images, acquired at 10 frames·s^{-1}, were treated with ImageJ (19, 20). The first frame showing mechanical contact between the two droplets was considered as the starting of the fusion event (t=0). In case of mobile droplets, the StackReg plugin (21) was used to correct for the drift of the images. Subsequently, a mask was applied to individual droplets, and their elliptical shape was tracked over time (Fig. S5(a)). Then, the aspect ratio, A.R., the ratio of the two axis of the obtained ellipse), was plotted against time and fitted with an exponential function:

$$A.R. = 1 + ke^{-\frac{t}{\tau}} \quad \text{(S2)}$$

where k=A.R.$_{(t=0)} - 1$. The characteristic fusion time (τ) was plotted against the average droplet length scale, defined as $\sqrt{(major\ axis_{t=0} - minor\ axis_{t=0}) \cdot minor\ axis_{t=0}}$ (Fig. S5(b)). Finally, data were fitted with a linear curve imposing the

intercept equal to 0, obtaining the slope (inverse capillary velocity) $\eta/\gamma = 1.1 \pm 0.2$ s·μm^{-1}. We monitored nine coalescence events, collected during two experimental sessions.

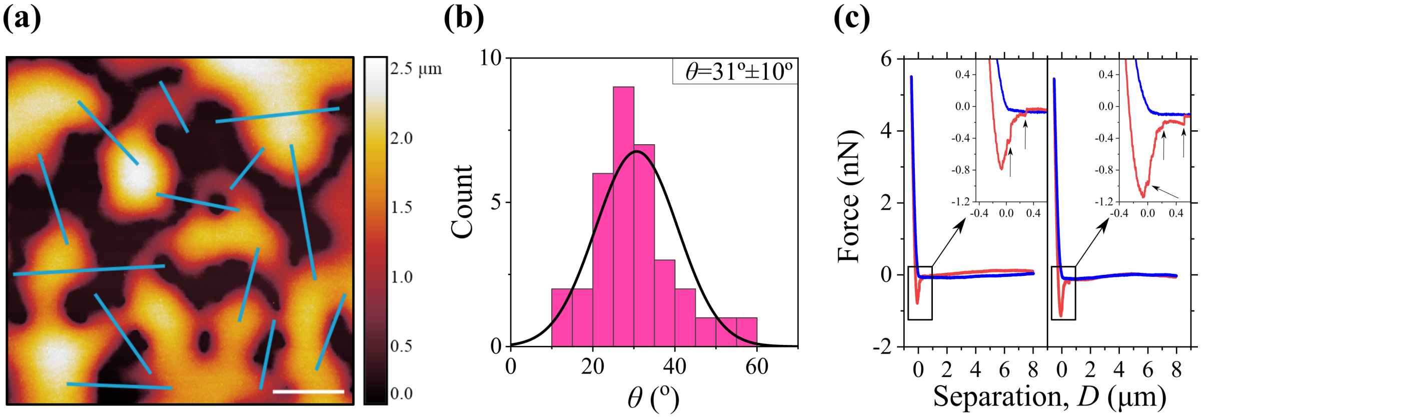

Figure S1: **(a)** AFM image (512 x 512 pixels) of droplets wetting large regions of a glass coverslip. Scale bar (white segment) = 10 μm. The profiles depicted in light blue (thickness 5 pixels) were taken into account to extract the contact angle θ. **(b)** Normal distribution of all the values obtained for θ. **(c)** Example of two indentation cycles on an aged (gel) droplet, exhibiting several force steps (arrows) while retracting the probe from the droplet and suggesting intermolecular ruptures. The blue curve is the approach, the red is the retract.

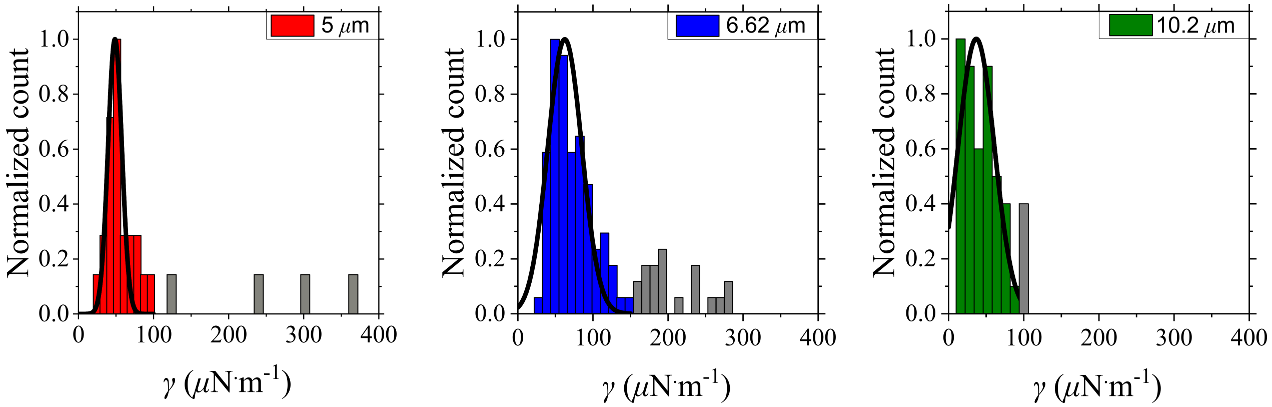

Figure S2: Surface tension distributions obtained with different AFM colloidal probe sizes. The black lines represent the associated Gaussian best fit distribution. Negligible secondary populations, probably due to condensate aging, were excluded from the fit (grey).

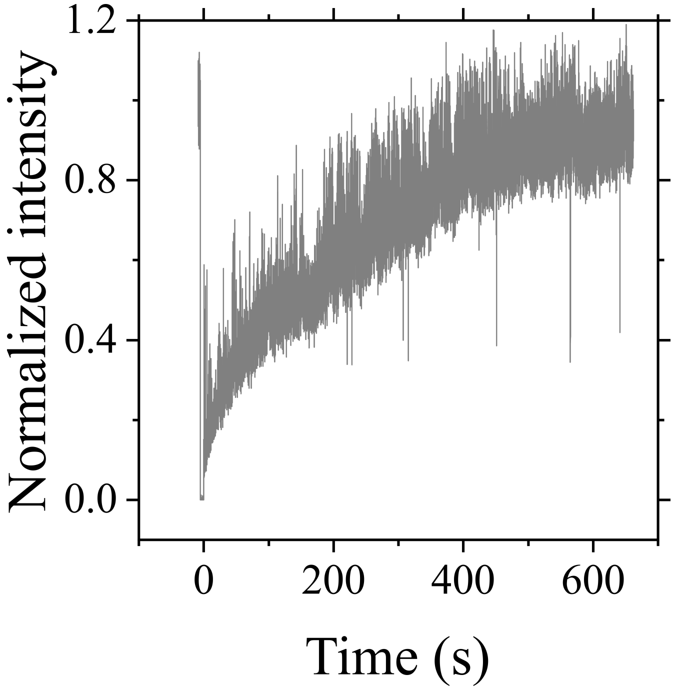

Figure S3: Recurrent FRAP dataset, showing complete recovery within a few minutes.

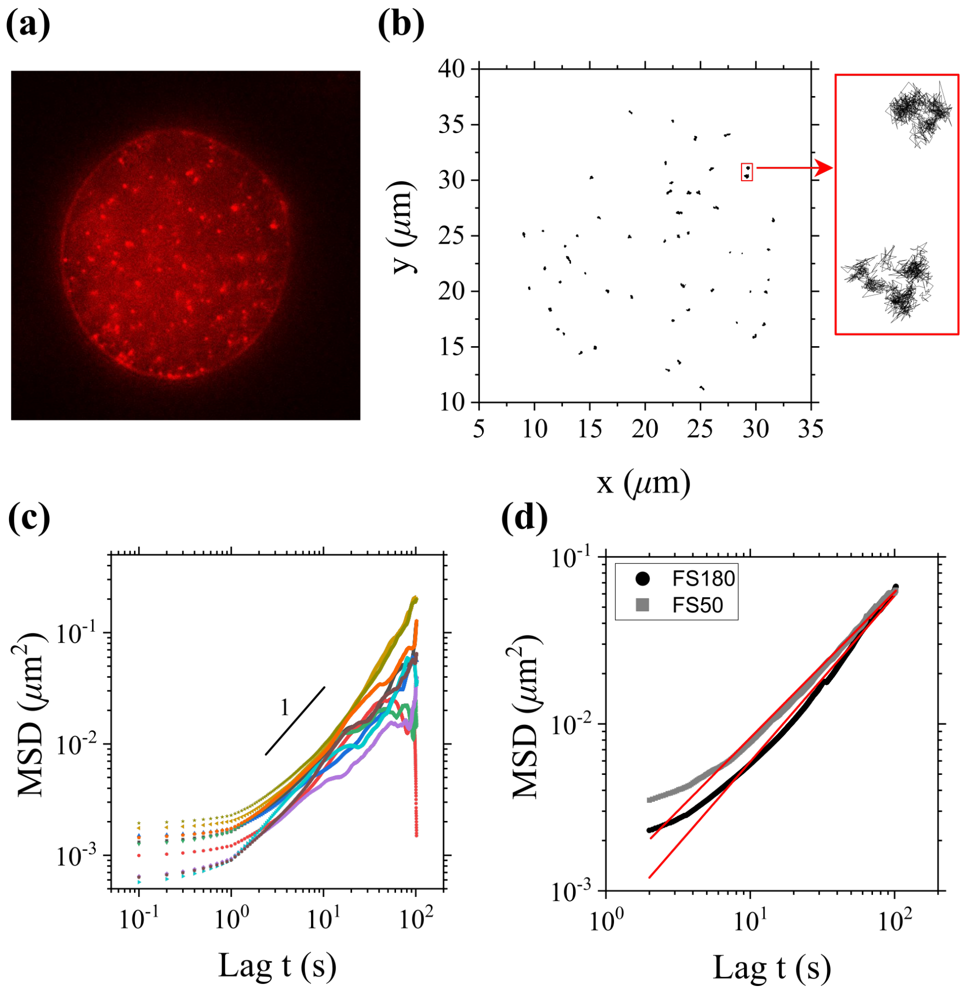

Figure S4: **(a)** 50 nm beads embedded into a droplet. Panel **(b)** shows several beads trajectories over 100 s, alongside with a zoom of two of these. **(c)** Examples of typical MSD curves for single 50 nm beads embedded within a condensate. **(d)** Averaged MSD curves for 50 nm and 180 nm fluorescent beads embedded in one liquid droplet, with associated fit.

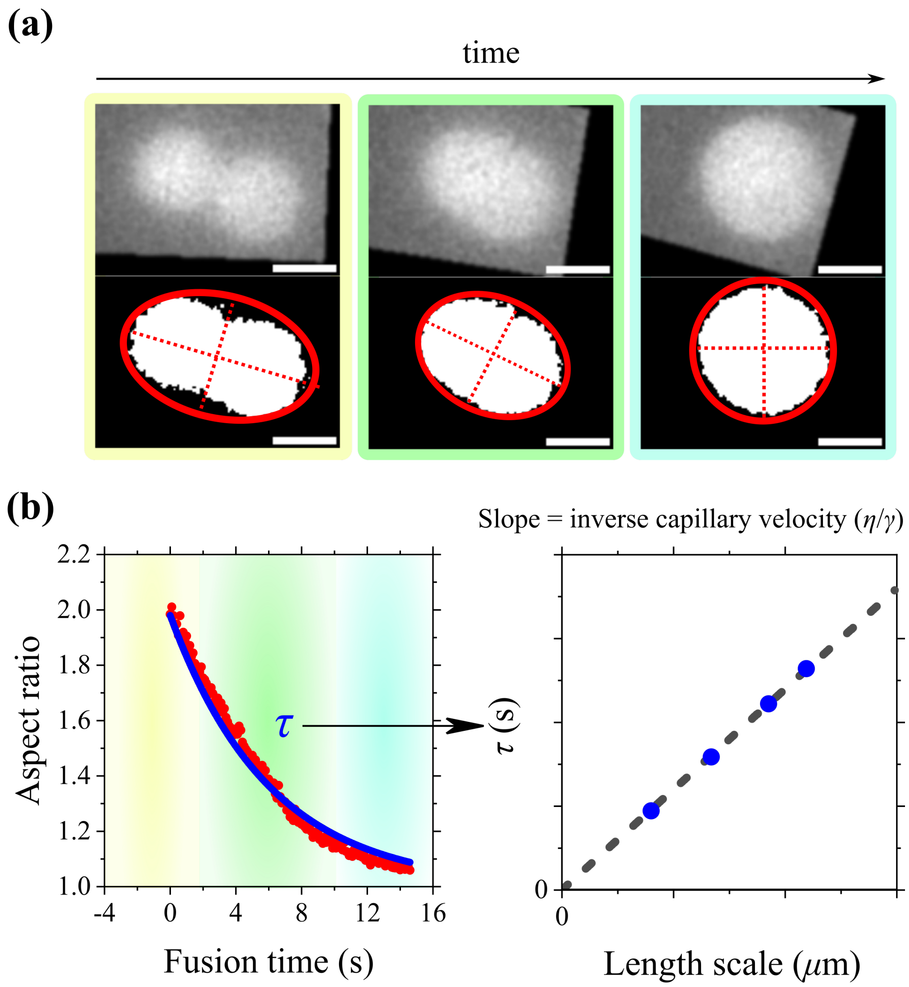

Figure S5: **(a)** Images reporting coalescence of two droplets, from first contact, t=0 (yellow panel), until complete fusion (cyan panel). Scale bars = 3 μm. The bottom panels show the mask applied to follow the fusion events, including the red ellipse and circle indicating the output of droplet segmentation (*Analyze particles* tool in ImageJ). The red dotted lines indicate the major and minor axis, used to calculate the aspect ratio. The data treatment was performed following the example of ref (22). **(b)** The left panel reports the aspect ratio plotted against fusion time (red circles) and the corresponding exponential fit (blue line). From the fit, the characteristic fusion time (τ) is determined. The right panel shows an ideal representation of the evaluation of the inverse capillary velocity (real data in Fig. 4 in main manuscript).

SUPPORTING REFERENCES

1. Hutin, S., J. R. Kumita, V. I. Strotmann, A. Dolata, W. L. Ling, N. Louafi, A. Popov, P.-E. Milhiet, M. Blackledge, M. H. Nanao, P. A. Wigge, Y. Stahl, L. Costa, M. D. Tully, and C. Zubieta, 2023. Phase separation and molecular ordering of the prion-like domain of the Arabidopsis thermosensory protein EARLY FLOWERING 3. Proc. National Acad. Sci. 120:e2304714120. https://www.pnas.org/doi/abs/10.1073/pnas.2304714120.

2. Jung, J. H., A. D. Barbosa, S. Hutin, J. R. Kumita, M. Gao, D. Derwort, C. S. Silva, X. Lai, E. Pierre, F. Geng, S. B. Kim, S. Baek, C. Zubieta, K. E. Jaeger, and P. A. Wigge, 2020. A prion-like domain in ELF3 functions as a thermosensor in Arabidopsis. Nature 585:256–260. https://doi.org/10.1038/s41586-020-2644-7.

3. Vial, A., C. Taveneau, L. Costa, B. Chauvin, H. Nasrallah, C. Godefroy, P. Dosset, H. Isambert, K. X. Ngo, S. Mangenot, D. Levy, A. Bertin, and P. E. Milhiet, 2021. Correlative AFM and fluorescence imaging demonstrate nanoscale membrane remodeling and ring-like and tubular structure formation by septins. Nanoscale 13:12484–12493. https://doi.org/10.1039/D1NR01978C.

4. Dahmane, S., C. Doucet, A. Le Gall, C. Chamontin, P. Dosset, F. Murcy, L. Fernandez, D. Salas, E. Rubinstein, M. Mougel, M. Nollmann, and P. E. Milhiet, 2019. Nanoscale organization of tetraspanins during HIV-1 budding by correlative dSTORM/AFM. Nanoscale 11:6036–6044. https://pubs.rsc.org/en/content/articlehtml/2019/nr/c8nr07269h.

5. Vial, A., L. Costa, P. Dosset, P. Rosso, G. Boutières, O. Faklaris, H. Haschke, P. E. Milhiet, and C. M. Doucet, 2023. Structure and mechanics of the human nuclear pore complex basket using correlative AFM-fluorescence superresolution microscopy. Nanoscale 15:5756–5770. https://doi.org/10.1039/D2NR06034E.

6. Elena-Real, C. A., A. Sagar, A. Urbanek, M. Popovic, A. Morató, A. Estaña, A. Fournet, C. Doucet, X. L. Lund, Z. D. Shi, L. Costa, A. Thureau, F. Allemand, R. E. Swenson, P. E. Milhiet, R. Crehuet, A. Barducci, J. Cortés, D. Sinnaeve, N. Sibille, and P. Bernadó, 2023. The structure of pathogenic huntingtin exon 1 defines the bases of its aggregation propensity. Nat. Struct. Mol. Biol. 2023 30:3 30:309–320. https://www.nature.com/articles/s41594-023-00920-0.

7. Schatz, M., L. Marty, C. Ounadjela, P. B. V. Tong, I. Cardace, C. Mettling, P.-E. Milhiet, L. Costa, C. Godefroy, M. Pugnière, J.-F. Guichou, J.-M. Mesnard, M. Blaise, and B. Beaumelle, 2023. A Tripartite Complex HIV-1 Tat-Cyclophilin A-Capsid Protein Enables Tat Encapsidation That Is Required for HIV-1 Infectivity. J. Virol. 97:e00278–23. https://doi.org/10.1128/jvi.00278-23.

8. Fernandes, T. F. D., O. Saavedra-Villanueva, E. Margeat, P.-E. Milhiet, and L. Costa, 2020. Synchronous, Crosstalk-free Correlative AFM and Confocal Microscopies/Spectroscopies. Sci. Reports 10:7098. https://doi.org/10.1038/s41598-020-62529-3.

9. Sader, J. E., R. Borgani, C. T. Gibson, D. B. Haviland, M. J. Higgins, J. I. Kilpatrick, J. Lu, P. Mulvaney, C. J. Shearer, A. D. Slattery, P. A. Thorén, J. Tran, H. Zhang, H. Zhang, and T. Zheng, 2016. A virtual instrument to standardise the calibration of atomic force microscope cantilevers. Review Sci. Instruments 87. https://doi.org/10.1063/1.4962866.

10. Proksch, R., T. E. Schäffer, J. P. Cleveland, R. C. Callahan, and M. B. Viani, 2004. Finite optical spot size and position corrections in thermal spring constant calibration. Nanotechnology 15:1344–1350. https://iopscience.iop.org/article/10.1088/0957-4484/15/9/039.

11. Nečas, D., and P. Klapetek, 2012. Gwyddion: An open-source software for SPM data analysis. Central Eur. J. Phys. 10:181–188. https://doi.org/10.2478/s11534-011-0096-2.

12. Dosset, P., P. Rassam, L. Fernandez, C. Espenel, E. Rubinstein, E. Margeat, and P. E. Milhiet, 2016. Automatic detection of diffusion modes within biological membranes using back-propagation neural network. BMC Bioinform. 17:1–12. http://dx.doi.org/10.1186/s12859-016-1064-z.

13. Qian, H., M. P. Sheetz, and E. L. Elson, 1991. Single particle tracking. Analysis of diffusion and flow in two-dimensional systems. Biophys. J. 60:910–921. http://dx.doi.org/10.1016/S0006-3495(91)82125-7.

14. Kusumi, A., Y. Sako, and M. Yamamoto, 1993. Confined lateral diffusion of membrane receptors as studied by single particle tracking (nanovid microscopy). Effects of calcium-induced differentiation in cultured epithelial cells. Biophys. J. 65:2021–2040. http://dx.doi.org/10.1016/S0006-3495(93)81253-0.

15. Garting, T., and A. Stradner, 2019. Synthesis and application of PEGylated tracer particles for measuring protein solution viscosities using Dynamic Light Scattering-based microrheology. Colloids Surfaces B: Biointerfaces 181:516–523. https://doi.org/10.1016/j.colsurfb.2019.05.059.

16. Daniels, B. R., B. C. Masi, and D. Wirtz, 2006. Probing single-cell micromechanics in vivo: The microrheology of C. elegans developing embryos. Biophys. J. 90:4712–4719. https://doi.org/10.1529/biophysj.105.080606.

17. Alshareedah, I., K. Taranpreet, and P. R. Banerjee, 2021. Methods for Characterizing the Material Properties of Biomolecular Condensates. Methods Enzymol. 646:143–183. https://doi.org/10.1016/bs.mie.2020.06.009.

18. Feric, M., and C. P. Brangwynne, 2013. A nuclear F-actin scaffold stabilizes RNP droplets against gravity in large cells. Nat. Cell Biol. 2012 14:5 15:1253–1259. https://doi.org/10.1038/ncb2830.

19. Schneider, C. A., W. S. Rasband, and K. W. Eliceiri, 2012. NIH Image to ImageJ: 25 years of image analysis. Nat. Methods 2012 9:7 9:671–675. https://www.nature.com/articles/nmeth.2089.

20. Abràmoff, M. D., P. J. Magalhães, and S. J. Ram, 2004. Image processing with imageJ. Biophotonics Int. 11:36–41.

21. Thévenaz, P., U. E. Ruttimann, and M. Unser, 1998. A pyramid approach to subpixel registration based on intensity. IEEE Trans. on Image Process. 7:27–41. https://doi.org/10.1109/83.650848.

22. Ceballos, A. V., C. J. McDonald, and S. Elbaum-Garfinkle, 2018. Methods and Strategies to Quantify Phase Separation of Disordered Proteins. Methods Enzymol. 611:31–50. https://doi.org/10.1016/bs.mie.2018.09.037.
